# CCR5-overexpressing mesenchymal stem cells protect against experimental autoimmune uveitis: insights from single-cell transcriptome analysis

**DOI:** 10.1186/s12974-024-03134-3

**Published:** 2024-05-27

**Authors:** Fa Yuan, Rong Zhang, Jiani Li, Qiannan Lei, Shuyi Wang, Fanying Jiang, Yanan Guo, Mengqing Xiang

**Affiliations:** 1 State Key Laboratory of Ophthalmology, Guangdong Provincial Key Laboratory of Ophthalmology and Visual Science, Zhongshan Ophthalmic Center, Sun Yat-sen University, Guangzhou, 510060 China; 2 Guangdong Provincial Key Laboratory of Brain Function and Disease, Zhongshan School of Medicine, Sun Yat-sen University, Guangzhou, 510080 China

**Keywords:** Experimental autoimmune uveitis, scRNA-seq, CCL5/CCR5, Mesenchymal stem cells

## Abstract

Autoimmune uveitis is a leading cause of severe vision loss, and animal models provide unique opportunities for studying its pathogenesis and therapeutic strategies. Here we employ scRNA-seq, RNA-seq and various molecular and cellular approaches to characterize mouse models of classical experimental autoimmune uveitis (EAU), revealing that EAU causes broad retinal neuron degeneration and marker downregulation, and that Müller glia may act as antigen-presenting cells. Moreover, EAU immune response is primarily driven by Th1 cells, and results in dramatic upregulation of CC chemokines, especially CCL5, in the EAU retina. Accordingly, overexpression of CCR5, a CCL5 receptor, in mesenchymal stem cells (MSCs) enhances their homing capacity and improves their immunomodulatory outcomes in preventing EAU, by reducing infiltrating T cells and activated microglia and suppressing Nlrp3 inflammasome activation. Taken together, our data not only provide valuable insights into the molecular characteristics of EAU but also open an avenue for innovative MSC-based therapy.

**Supplementary Information:**

The online version contains supplementary material available at 10.1186/s12974-024-03134-3.

## Introduction

Uveitis is a major sight-threatening ocular inflammatory disease worldwide, whose sequelae include cataracts, glaucoma, vitreous opacities, retinal detachment, and retinal vascular abnormalities, etc [1–5]. Autoimmune uveitis, one kind of uveitis, is estimated to be the fourth leading cause of severe vision loss in the industrialized world [4] and occurs in a variety of diseases [6]. Experimental autoimmune uveitis (EAU) induced by the interphotoreceptor retinoid-binding protein (IRBP), which shares many pathological characteristics with those of human uveitis, is widely used to help illuminate the genetic influences on, resolve the pathogenic mechanisms of, and test potential therapeutic paradigms for human uveitis since 1988 [7–9]. However, no single animal model such as EAU can reproduce the full spectrum of features of the human disease. To better understand the pathogenesis of the disease, in addition to the induced model of autoimmune uveitis, two spontaneous uveitis models, R161H and *Aire*^*−/−*^ mice, were also established subsequently [10, 11]. The classical EAU induced by IRBP is Th17- or Th1-dependent [12–15], while the two spontaneous uveitis models, which rely on genetic backgrounds, have been reported to be IFNγ- and Th1-dependent, respectively [12, 16].

Previous study suggests that EAU is accompanied by blood-retinal barrier (BRB) breakdown [17]. Meanwhile, Crane et al. reported that CCR5, a chemokine receptor, plays an important migratory role for cells entering the BRB and retina [18]. It has been suggested that EAU triggers the expression and release of chemokines, which in turn facilitate the trans-endothelial migration of immune cells and mediate the BRB disruption. Nevertheless, *CCR5* deletion failed to prevent the development of EAU, although it did reduce T-cell infiltration into the eye [19]. It has also been speculated that the chemokine ligands mediate the trafficking of mesenchymal stem cells (MSCs) and their infiltration of the injured tissue [20]. However, isolated MSCs were found to gradually lose their homing capacity to the targeted lesions during continuous passage [21–23]. Therefore, strategies for enhancing the homing ability of infused MSCs to the impaired retinas may benefit therapeutic treatments.

Although single-cell RNA sequencing (scRNA-seq) analysis has been used to characterize the cell types and gene expression patterns in *Aire*^*−/−*^ mouse retinas [16], the classical EAU model induced by IRBP has not yet been characterized using this powerful and unbiased method. To investigate the pathogenesis and therapeutic strategy of uveitis, we characterized the neural retinas of EAU mice on a C57BL/6 background using both scRNA-seq and bulk RNA-seq analyses in the present study. We found that EAU caused a decrease in marker expression levels and cell number of all retinal neuron types, and that Müller glia might act as antigen-presenting cells (APCs) during this process. Moreover, the classical EAU induced by IRBP peptide 651–670 was Th1-dependent, and caused dramatic upregulation of the chemokine CCL5 in the EAU retinas. Based on these observations, we speculated that MSCs overexpressing human CCR5, a CCL5 receptor, would have enhanced homing capacity and better attenuate EAU through their immunomodulatory capacity than regular MSCs. And indeed, we were able to show that CCR5-overexpressing MSCs exhibited a better therapeutic effect on EAU than regular MSCs, hence providing a novel MSC-based therapy option.

## Results

### Diverse immune cells infiltrate into the retina of EAU mice

**Fig. 1:**
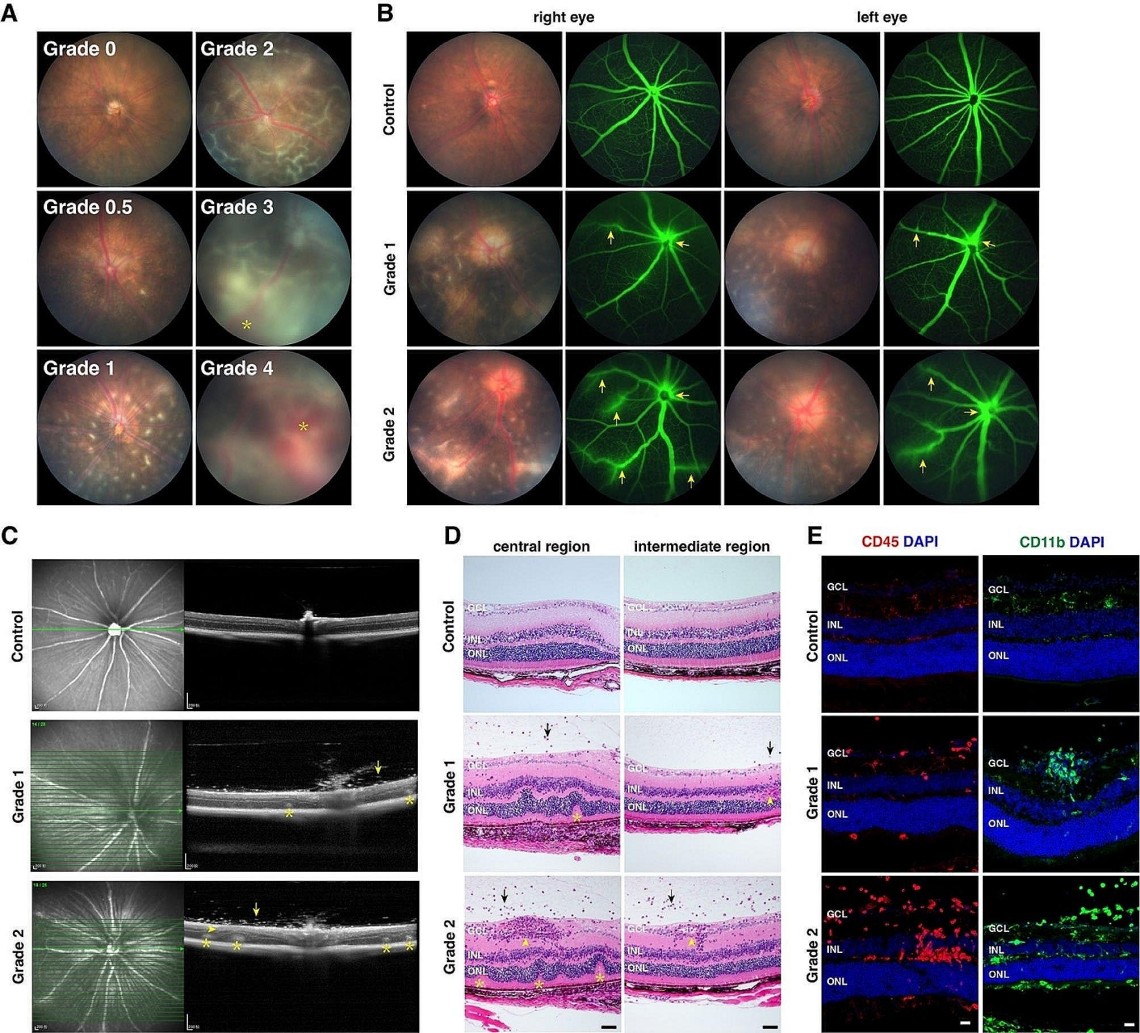
Characterization of EAU retinas. (**A**) Representative fundus photographs (14 d.p.i.) showing the range of disease severity and corresponding grades. The asterisks indicate subretinal hemorrhages. (**B**) Fundus photographs and corresponding fluorescein angiograms (21 d.p.i.) of EAU eyes. The arrows indicate areas of fluorescein leakage. (**C**) Fundus photographs (left) and OCT images (right) (14 d.p.i.) showing inflammatory infiltrating cells (arrows), vasculitis (arrowhead), and retinal folds and detachment (asterisks) in EAU eyes. (**D**) Hematoxylin-eosin staining (14 d.p.i.) revealed inflammatory infiltrating cells (arrows), vasculitis (arrowheads), and retinal folds and detachment (asterisks) in EAU eyes. (**E**) EAU increased leukocytes immunoreactive for CD45 and myeloid cells immunoreactive for CD11b in the retinas (14 d.p.i.). All retinal sections were counterstained with nuclear DAPI. GCL: ganglion cell layer; INL: inner nuclear layer; ONL: outer nuclear layer. Scale bar: (**D**, **E**) 20 μm

As a first step to characterize retinal changes in single-cell transcriptome caused by EAU, we established EAU mouse models by immunization with a human IRBP peptide 651–670 as described [24]. To confirm these mouse models, we subsequently performed fundus imaging, fluorescein fundus angiography (FFA), and optical coherence tomography (OCT) on mice every 7 days postimmunization (d.p.i.) to grade disease severity according to the published clinical grading scale [24, 25] (Table S1). The incidence rate was 89.4% ± 7.9% at 21 d.p.i. Figure 1A, B shows representative fundus images and fluorescein angiograms for the different clinical grades (grades 0–4). In general, in the eyes of EAU mice from low to high grades, there was progressive vascular leakage (Fig. 1A, B), retinal disorganization (Fig. 1C, D), and increase in the number of infiltrating CD45-positive leukocytes and CD11b-positive myeloid cells (Fig. 1C-E), consistent with previous observations [9, 24–27].

### Establishing the cell atlas for EAU retinas based on single-cell RNA-Seq

To elucidate changes in the composition and number of retinal cells when EAU arose, we used mice at grade 2 (14 d.p.i.) and their wild-type littermates for scRNA-seq analysis (Fig. S1). We performed scRNA-seq profiling using the 10X Genomics platform to obtain 20,448 single cells from 8 retinas: 2 grade 2 mice (10,369 cells) and 2 wild-type controls (10,079 cells). A total of 147 presumed doublet cells were excluded from subsequent analysis.

**Fig. 2:**
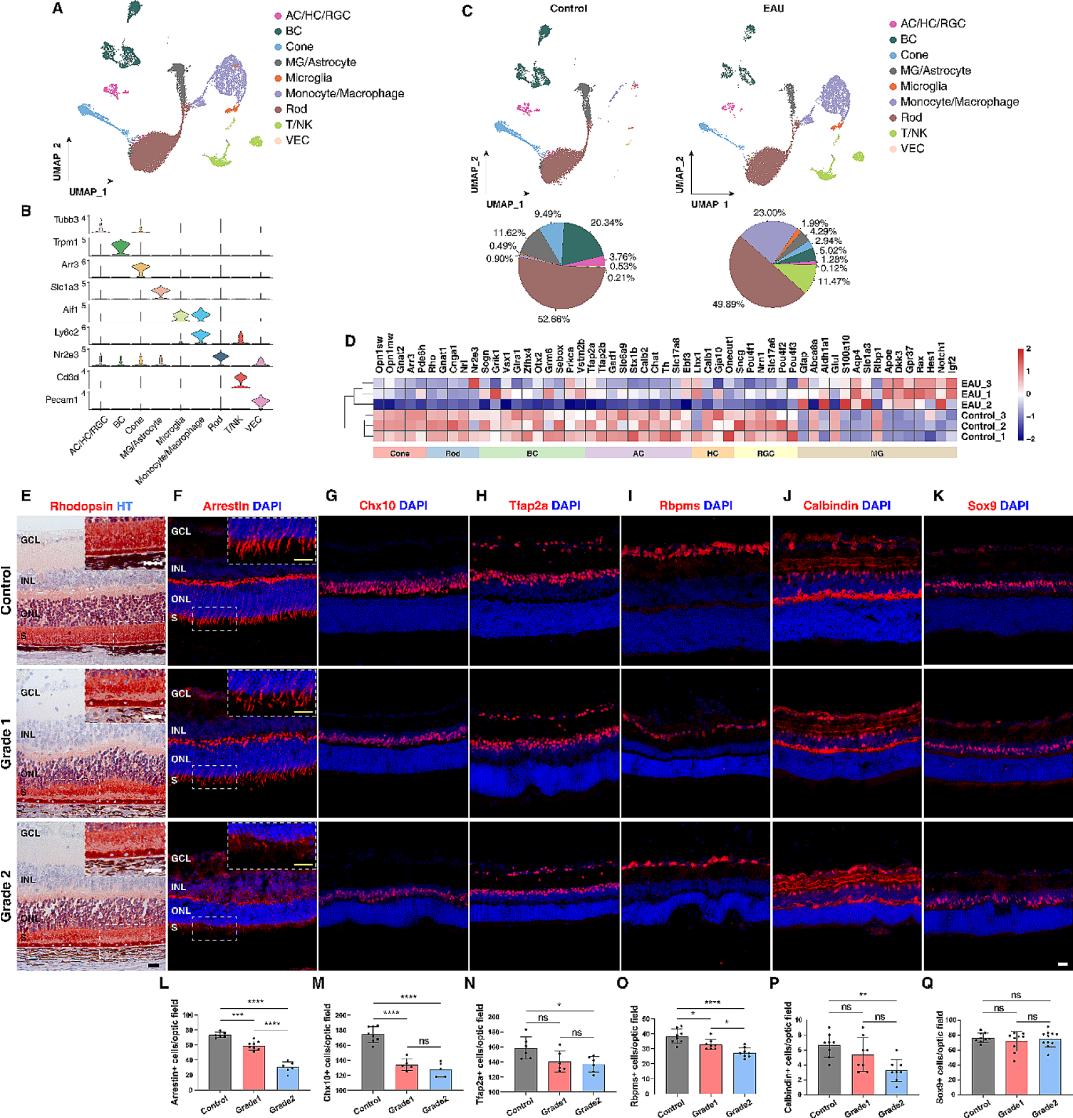
Single-cell transcriptome atlas and neuron loss in EAU retinas. (**A**) UMAP plot showing different cell-type clusters in a merged dataset from duplicate EAU and control wild-type retinas. (**B**) Stacked violin plots showing expression patterns of representative cell type marker genes in different cell clusters. (**C**) UMAP plots and pie charts comparing the cell types in control and EAU retinas. (**D**) Heatmap of expression levels of a set of marker genes for cone, rod, bipolar, amacrine, horizontal, retinal ganglion, or Müller glial cells in control and EAU retinas, determined by bulk RNA-seq analysis. (**E**-**K**) Immunostaining of retinal sections for the indicated cell type-specific protein markers showed obvert reduction of retinal neurons after EAU onset. All retinal sections were also counterstained with nuclear DAPI or hematoxylin (HT). (**L**-**Q**) Quantification of cells immunoreactive for the indicated protein markers in control and EAU retinas. Data are presented as mean ± SD (*n* = 4–11 retinas per group). *p < 0.05, **p < 0.01, ***p < 0.001, ****p < 0.0001, ns, no significance. AC: amacrine cell; BC: bipolar cell; GCL: ganglion cell layer; HC: horizontal cell; INL: inner nuclear layer; MG: Müller glia; NK: natural killer cell; ONL: outer nuclear layer; RGC: retinal ganglion cell; S: inner and outer segments; T: T cell; VEC: vascular endothelial cell. Scale bar: (**E**-**K**) 20 μm

Combining Seurat [28, 29] clustering analysis with known molecular markers for various cell types (Fig. 2B; Tables S2, S3), we segregated the single cells into 9 major cell clusters based on cell types: immune cells [Microglia, Monocyte/Macrophage, and T/NK (T cell/natural killer cell)], retinal cells [AC/HC/RGC (amacrine cell/horizontal cell/retinal ganglion cell), BC (bipolar cell), Cone, MG (Müller glia)/Astrocyte, and Rod], and vascular endothelial cells (VEC) (Fig. 2A). Comparison of control and EAU cell clusters revealed a decrease in the number of multiple neuronal types in EAU retinas, especially in the BC cluster (20.34–5.02%), Cone cluster (9.49–2.94%), and AC/HC/RGC cluster (3.76–1.28%) (Fig. 2C). In agreement, bulk RNA-seq analysis showed that in EAU retinas, there was downregulation of marker genes for all 6 retinal neuron types including rods, cones, BCs, ACs, HCs, and RGCs (Fig. 2D; S2A, B). Immunostaining analysis further validated these results. In EAU retinas, there was obvious degeneration of rhodopsin^+^ rod inner and outer segments (Fig. 2E), and in general, progressive loss of Arrestin^+^ cones, Chx10^+^ BCs, Tfap2a^+^ ACs, Rbpms^+^ RGCs, and Calbindin^+^ HCs (Fig. 2F-J, L-P). By contrast, EAU did not appear to significantly alter Sox9^+^ Müller glia although resulting in an upregulation of their marker genes (Fig. 2K, Q). Therefore, EAU may cause not only progressive degeneration of all retinal neuron types but also a switch in the expression profile of Müller glia.

### The Müller glia may function as antigen-presenting cells

Müller glia are the major glial component of the retina, which are specified from the retinal progenitor cells [30]. Their processes act as the scaffold of the neural retina, ramifying throughout the whole neural retina between the nerve fiber layer and the outer limiting membrane. Furthermore, Müller glia are part of the blood-retinal barrier and are thought to contribute to ocular immune privilege by inhibiting the proliferation and activation of lymphocytes [31]. Thus, immune cells infiltrating into the retina must necessarily pass and come into contact with Müller glia.

**Fig. 3:**
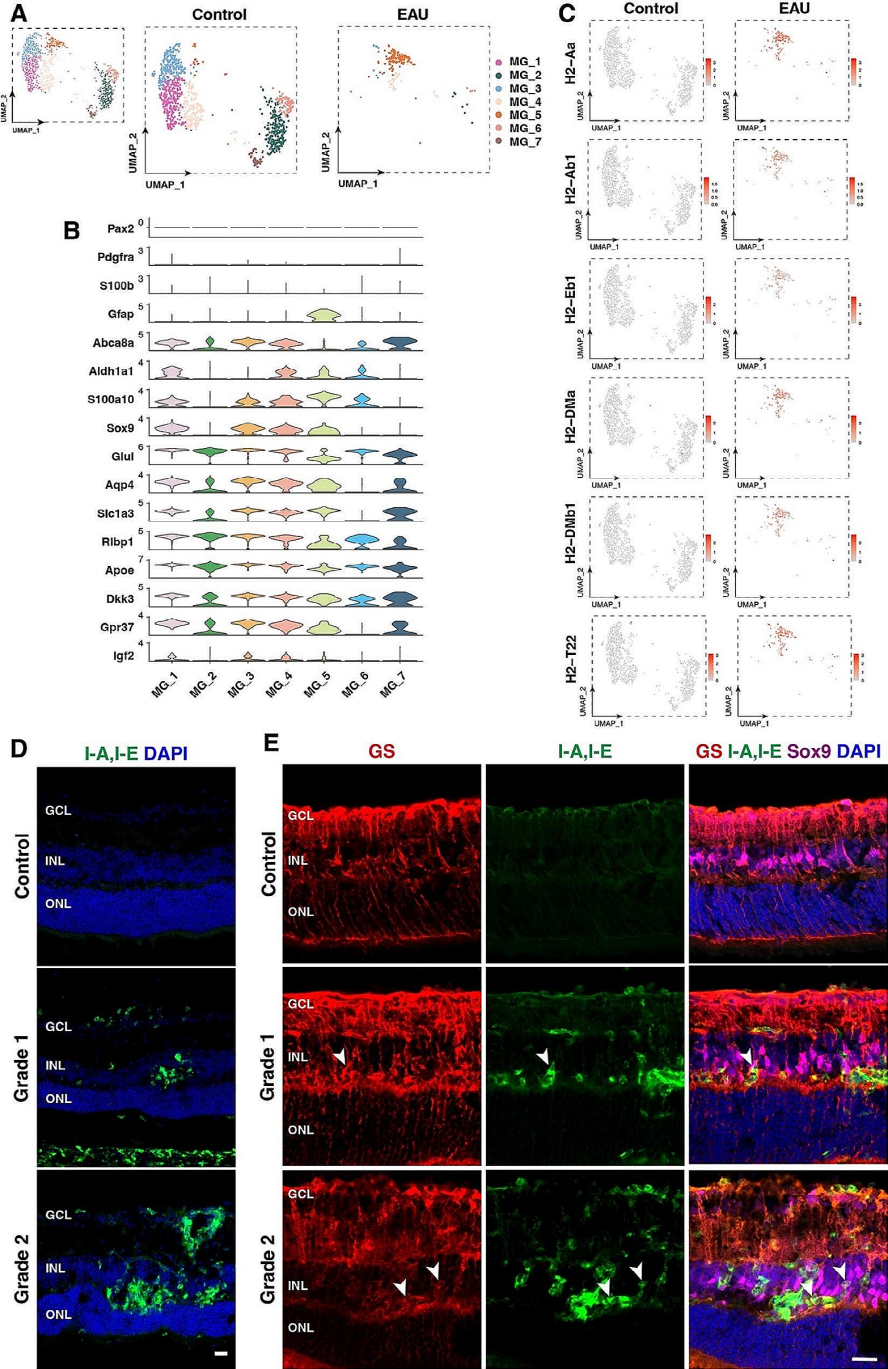
Upregulation of MHC II genes in Müller glia of EAU retinas. (**A**) UMAP plots of Müller glia clusters in control and EAU retinas. (**B**) Stacked violin plots showing expression patterns of known Müller glia and astrocyte marker genes in different Müller cell clusters. (**C**) Feature plots comparing the expression of MHC II genes in control and EAU retinas. (**D**) Immunostaining of control and EAU retinal sections for MHC II proteins I-A/I-E with DAPI counter-labeling. (**E**) Triple-immunostaining of control and EAU retinal sections for GS (glutamine synthetase, red), MHC II proteins (I-A/I-E) (green), and Sox9 (magenta) with DAPI counter-labeling (blue). Arrowheads indicate representative GS^+^Sox9^+^MHC II^+^ triple-positive Müller glia. GCL: ganglion cell layer; INL: inner nuclear layer; ONL: outer nuclear layer. Scale bars: (**D**, **E**) 20 μm

To investigate the effect of EAU on Müller glia, we re-analyzed the MG/Astrocyte cell cluster by UMAP clustering, which yielded 7 Müller glia clusters (MG_1–7) when astrocytes were removed (Figs. 2A and 3A). These Müller glia clusters exhibited distinct expression patterns of Müller cell marker genes (Fig. 3B; S3A), and of all 7 Müller glia clusters, MG_5, which was present mainly in EAU retinas (Fig. 3A), expressed *Gfap* (Fig. 3B), suggesting that most Müller glia acquired a reactive state following EAU. Intriguingly, the reactive Müller glia in MG_5 upregulated the major histocompatibility complex class II (MHC II) genes including *H2-Ab1* and *H2-Eb1* (Fig. 3C), which are mostly expressed by professional APCs [32, 33]. In agreement, increasing immunoreactivity for MHC II I-A/E alloantigens was detected in EAU retinas with increasing disease grades (Fig. 3D). Moreover, the MHC class II proteins were found to be enriched in Müller glial cell bodies (Fig. 3E), suggesting that Müller glia may function as non-professional APCs in EAU retinas.

Further analysis of our single-cell sequencing data showed only little or no expression of MHC II-related genes in amacrine cells, horizontal cells, RGCs, bipolar cells, rods, and cones of EAU retinas. However, their expression was detected in the microglia, MG/astrocyte, monocyte/macrophage, and VEC clusters (Fig. S3B). In agreement, immunostaining of EAU retinal sections revealed that some Cd31^+^ VECs, Pax2^+^ astrocytes and Sox9^+^ Müller glia expressed the MHC II proteins while there was no MHC II protein expression in Tfap2a^+^ amacrine cells, Rbpms^+^ RGCs, and Chx10^+^ bipolar cells (Fig. S3C-H).

### T cell diversity in the EAU retina

**Fig. 4:**
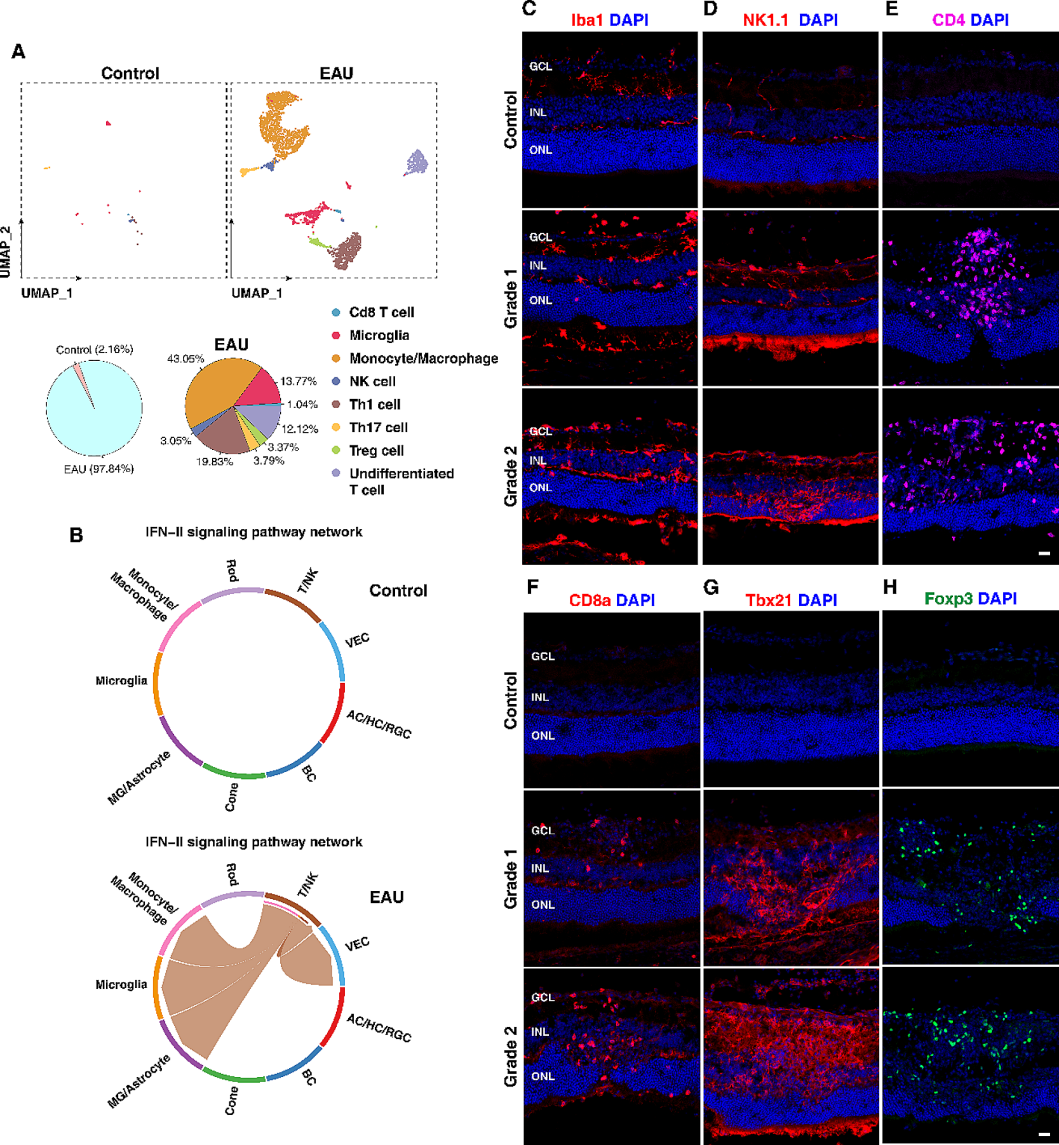
The diversity of immune cells in EAU retinas. (**A**) UMAP plots and pie charts of immune cell clusters in control and EAU retinas. The left pie chart shows there are few immune cells in control retinas while the right pie chart shows that Th1 cell is the predominant class of T helper cells in EAU retinas. (**B**) Chord diagrams comparing the IFNγ (IFN II) signaling pathway in control and EAU retinas. Edge color denotes the signaling source. Segments with large arrows represent signaling targets and the inner bar represents signaling source in which the colors indicate signaling targets. (**C**-**H**) Immunostaining of control and EAU retinal sections with antibodies against the indicated immune cell markers with DAPI counter-labeling. GCL: ganglion cell layer; INL: inner nuclear layer; ONL: outer nuclear layer. Scale bar: (**C**-**H**) 20 μm

To determine the effect of EAU on immune cells, we re-analyzed all immune cell-related clusters (Microglia, Monocyte/Macrophage and T/NK) by UMAP clustering (Figs. 2A and 4A). By classifying these cells according to the known immune cell markers (Table S3), we identified 5 subtypes of T cells: Cd8a T cells, Th1 cells, Th17 cells, regulatory T (Treg) cells, and undifferentiated T cells (Cd4^+^). Th1 cells were the predominant class of T helper cells in EAU retinas, accounting for 19.83% of all immune cells (Fig. 4A). Immunostaining of EAU retinas verified the abundant presence of Cd8a^+^ T cells, Tbx21^+^ Th1 cells, Foxp3^+^ Treg cells, and Cd4^+^ undifferentiated T cells, in addition to Iba1^+^ microglia and NK1.1^+^ NK cells, which were absent or few in control retinas (Fig. 4C-H).

Th1 and Th17 cells are known to mediate interferon-gamma (IFN-II or IFNγ) signaling and IL17A signaling, respectively [34, 35]. Further examination of the scRNA-seq data using cell-cell communication analysis (CellChat [36]) revealed an increase of IFN-II signaling but not IL17A signaling in EAU retinas (Fig. 4B), consistent with Th1 cells as the predominant class of T helper cells present in EAU retinas.

### Increased expression of CC chemokines in EAU retinas

**Fig. 5:**
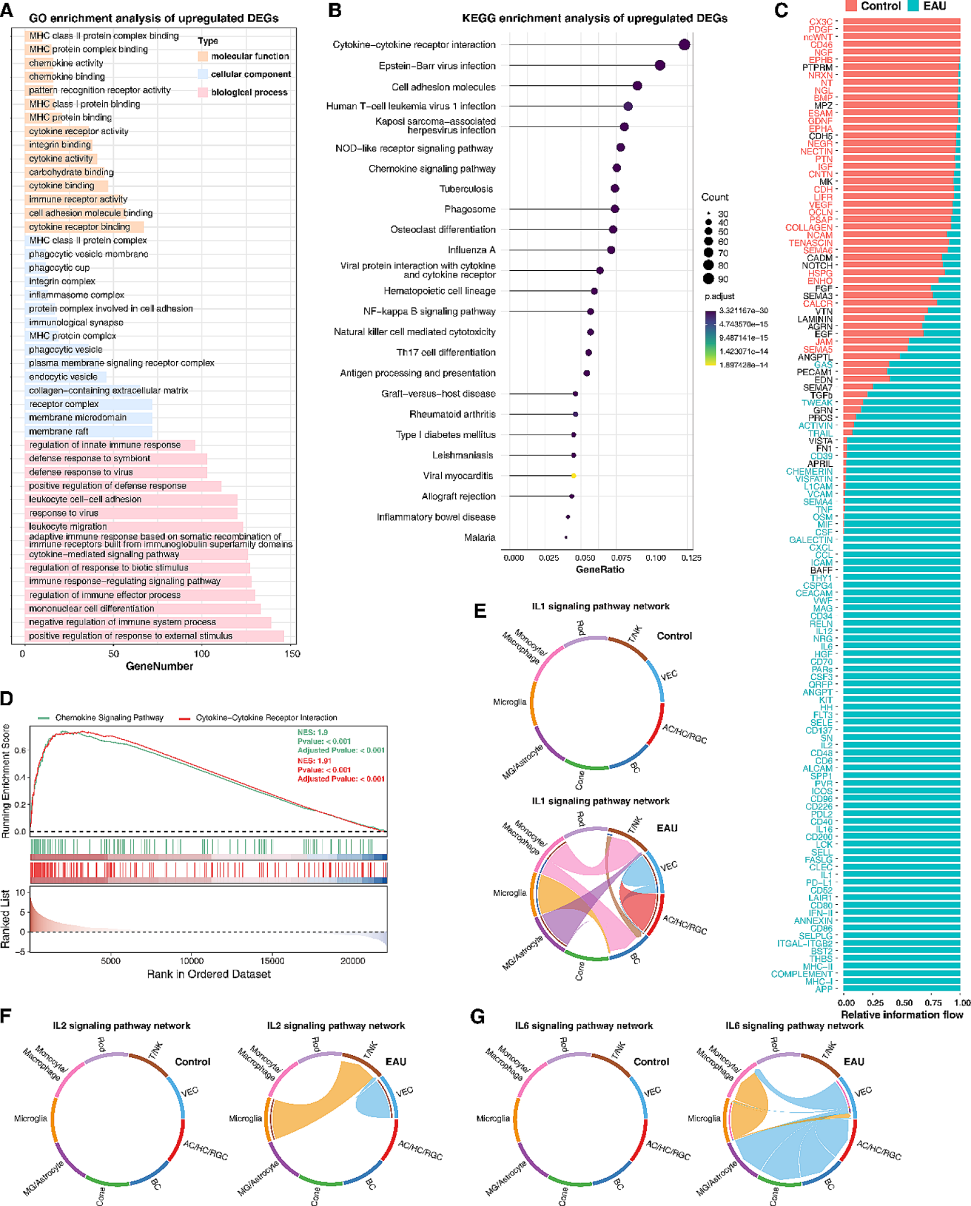
Enrichment of cytokine signaling pathways in EAU retinas. (**A**) GO enrichment analysis of the upregulated DEGs between EAU and control retinas, which were identified by bulk RNA-seq analysis. Shown are 15 top enriched GO terms each for molecular function, cellular component and biological process. (**B**) KEGG enrichment analysis of the upregulated DEGs between EAU and control retinas. (**C**) Barplot showing the indicated signaling pathways ranked based on their differences in overall information flow within the inferred networks. The top signaling pathways (shown in red) were more enriched in control retinas, whereas the bottom ones (shown in green) were more enriched in EAU retinas. (**D**) GSEA enrichment analysis of the bulk RNA-seq data identified enriched gene sets associated with the chemokine signaling pathway or cytokine-cytokine receptor interaction. (**E**-**G**) Chord diagrams comparing IL1 (**E**), IL2 (**F**) and IL6 (**G**) signaling pathways in control and EAU retinas. Edge color denotes the signaling source. Segments with large arrows represent signaling targets and inner bars represent signaling sources in which the colors indicate signaling targets

**Fig. 6:**
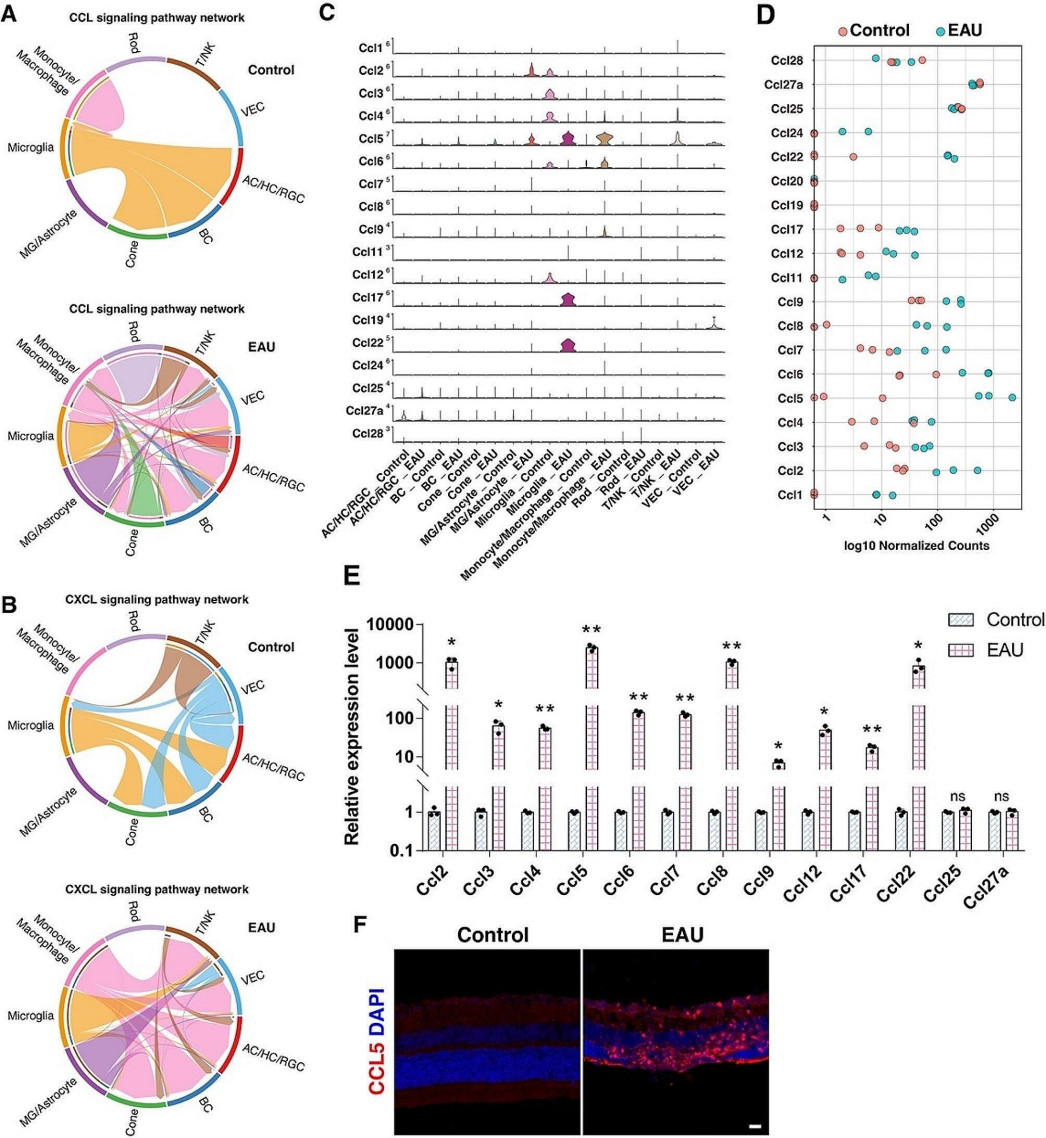
Chemokine signaling pathways were enriched in EAU retinas. (**A**, **B**) Chord diagrams comparing CC chemokine signaling pathway (**A**) and CXC chemokine signaling pathway (**B**) in control and EAU retinas. Edge color denotes the signaling source. Segments with large arrows represent signaling targets and inner bars represent signaling sources in which the colors indicate signaling targets. (**C**) Stacked violin plots showing the expression patterns of CC chemokine genes in control and EAU retinas based on scRNA-seq analysis. (**D**) Dot plot showing the expression of CC chemokine genes in control and EAU retinas based on bulk RNA-seq analysis. (**E**) qRT-PCR analysis showing the relative expression levels of the indicated CC chemokine genes in control and EAU retinas at 14 d.p.i. Data are presented as mean ± SD (*n* = 3 mice per group). *p < 0.05, **p < 0.01, ns, no significance. (**F**) Immunostaining of control and EAU retinal sections with an anti-CCL5 antibody with DAPI counter-labeling. Scale bar: (**F**) 20 μm

To further ascertain the molecular changes during EAU, we performed GO enrichment analysis of the significantly upregulated differentially expressed genes (DEGs) in EAU retinas, which were identified by bulk RNA-seq analysis (Fig. S2A, B). The enriched GO terms included MHC protein complex binding, MHC class II protein complex, chemokine activity, cytokine receptor activity, immune receptor activity, regulation of immune effector process, mononuclear cell differentiation, leukocyte cell-cell adhesion and migration, inflammasome complex, and so on (Fig. 5A), indicating increased immune response in the EAU retina. Moreover, KEGG enrichment analysis of the upregulated DEGs revealed that the most enriched pathway was cytokine-cytokine receptor interaction (Fig. 5B). And cell-cell communication analysis of the scRNA-seq data also revealed that a number of cytokine signaling pathways were enriched in EAU retinas compared to controls, such as IFN-II, IL1, IL2, IL6, CC chemokine, and CXC chemokine pathways (Figs. 4B, 5C and E-G and 6A and B). Consistent with this, GSEA for KEGG and Reactome enrichment analyses of the bulk RNA-seq data yielded enriched gene sets associated with chemokine signaling pathways and cytokine-cytokine receptor interaction (Fig. 5D; S2C), suggesting elevated chemokine signaling as one of the strong immune responses elicited by EAU.

Chemokines, a subclass of cytokines, are known to direct the migration of T cells [37]. As mentioned above, multiple subtypes of T cells infiltrated EAU retinas, so conceivably, chemokines may play a crucial role in this process. Cell-cell communication analysis of the scRNA-seq data showed that CCL signaling displayed a much more dramatic strength alteration than CXCL, CX3C or XC signaling between EAU and control retinas in Monocyte/Macrophage, T/NK, Microglia, and MG/Astrocyte cell clusters (Figure S4). Therefore, we further examined and compared the expression levels of CC chemokine family members in control and EAU retinas by analyzing the scRNA-seq and bulk RNA-seq datasets. This revealed significant upregulation in expression of about a dozen CC chemokine family members including *Ccl1-9, Ccl11, Ccl12, Ccl17, Ccl22, and Ccl24* (Fig. 6C, D). This result was subsequently validated by qRT-PCR assay (Fig. 6E). In particular, among all these chemokine genes, the expression level of *Ccl5* exhibited the most dramatic upregulation postimmunization, reaching over 2500-fold when compared to the control retinas (Fig. 6C-E). This may explain why Th1 cells were the most abundant T helper cells infiltrating into EAU retinas (Fig. 4A), given the fact that Th1 cells predominantly express CCR5, a receptor of CCL5 [38–41].

### CCR5-overexpressing MSCs increase migrative capacity towards CCL5 both in vitro and in vivo

Since MSCs have multiple regulatory effects on immunity [42–44] including EAU [45–47] and the chemokine-chemokine receptor axis is indispensable for MSC migration [23, 48], we sought to generate CCR5-overexpressing MSCs to enhance the homing capacity and therapeutic effects of MSCs (Fig. S5). First, we induced highly homogeneous MSCs from hiPSCs, using an efficient and chemically defined method [49] (Fig. S5C). Flow cytometric analysis showed that the hiPSC-derived MSCs expressed several typical MSC markers: CD29, CD44, CD73, and CD166, but lacked the expression of hematopoietic cell markers CD34 and CD45 (Fig. S5E). To test the multilineage differentiation potential of MSCs, we subjected them to differentiation procedures in adipogenic, osteogenic or chondrogenic differentiation media. Subsequent staining by alizarin red S, oil red O and toluidine blue confirmed that the MSCs had osteogenic, adipogenic and chondrogenic capacity, respectively (Fig. S5D). We then infected MSCs with lentiviruses expressing both CCR5 and tdTomato (referred to as MSC^CCR5^) or tdTomato only (referred to as MSC^tdTomato^), followed by purification of tdTomato-positive MSC^CCR5^ or MSC^tdTomato^ cells by fluorescence-activated cell sorting (FACS) (Fig. S5A-C, F). FACS-enriched MSC^CCR5^ cells were further shown to have similar multipotency as regular MSCs (Fig. S5G).

**Fig. 7:**
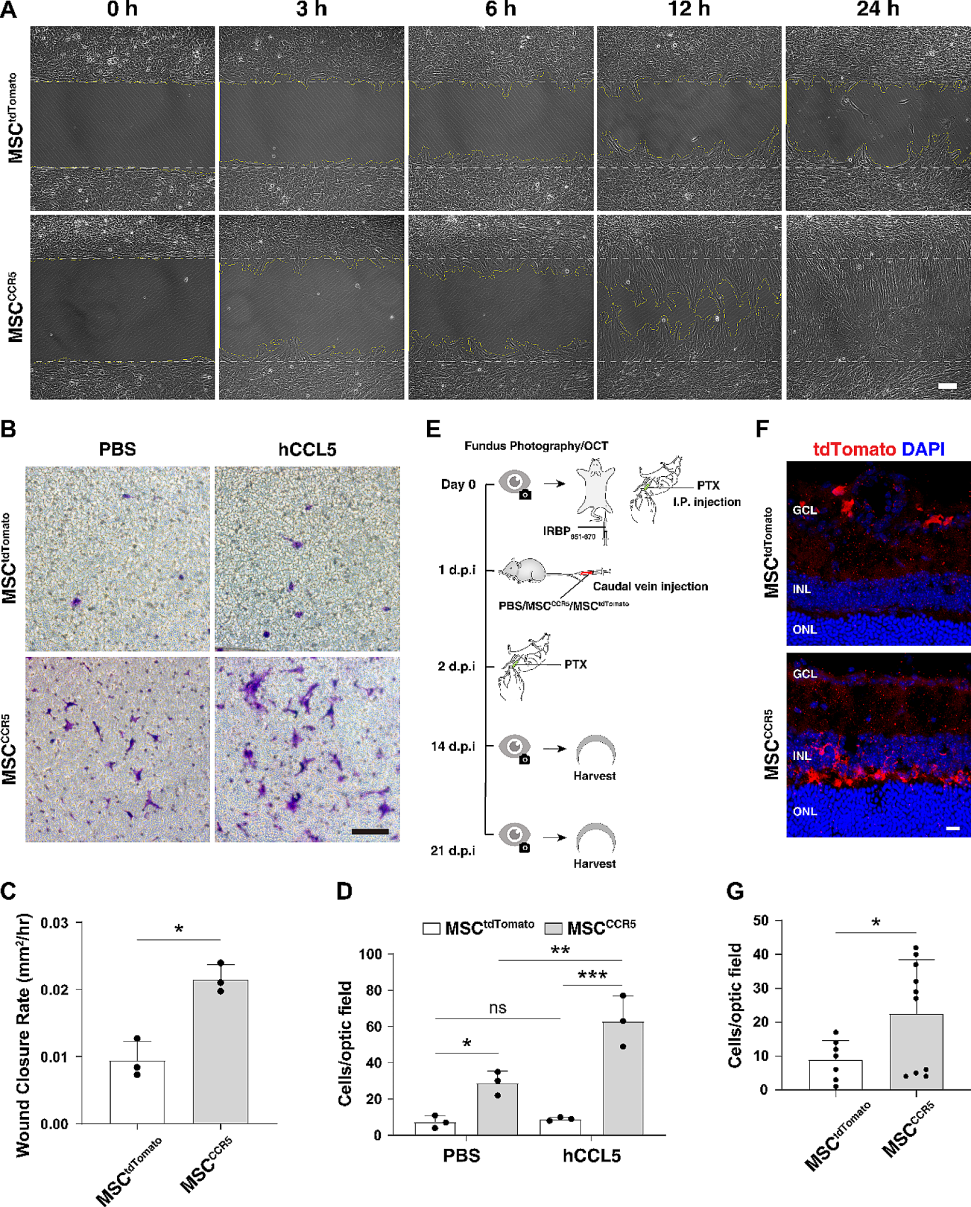
CCR5-overexpressing MSCs possess increased migrative capacity towards CCL5 in vitro and to EAU retinas in vivo. (**A**) Two-dimensional wound healing test was performed to detect the migration of MSC^tdTomato^ and MSC^CCR5^ cells toward hCCL5. Shown are phase-contrast images of the cell culture at the indicated timepoints following the removal of culture-insert. (**B**) Transwell migration assay was performed to detect the migration of MSC^tdTomato^ and MSC^CCR5^ cells toward hCCL5. MSCs migrated to the lower chamber surface were stained with crystal violet. (**C**) Quantification of gap closure in the wound healing test showed a significant increase in wound closure rate for MSC^CCR5^ cells compared to MSC^tdTomato^ cells. Data are presented as mean ± SD (*n* = 3 individual samples per group). *p < 0.01. (**D**) Quantification of migrated cells in the transwell migration assay showed a significant increase for MSC^CCR5^ cells compared to MSC^tdTomato^ cells. Data are presented as mean ± SD (*n* = 3 individual samples per group). *p < 0.05, **p < 0.01, ***p < 0.001, ns, no significance. (**E**) Schematic diagram of EAU induction and MSC transplantation into EAU mice. (**F**) Confocal images of transplanted tdTomato-positive MSCs located in the retina at 13 days post-injection. (**G**) Quantification of tdTomato-positive cells in retinas from mice transplanted with MSC^CCR5^ or MSC^tdTomato^ cells. Data are presented as mean ± SD (*n* = 7–10 retinas per group). *p < 0.05. d.p.i.: day(s) postimmunization; GCL: ganglion cell layer; INL: inner nuclear layer; IRBP: interphotoreceptor retinoid-binding protein; ONL: outer nuclear layer; PTX: pertussis toxin. Scale bar: (A, B) 100 μm, (**F**), 20 μm

To determine the homing capacity of CCR5-overexpressing MSCs, we first examined the migration of MSC^CCR5^ cells towards recombinant human CCL5 (hCCL5) through wound healing test (two-dimension) and transwell migration assay (three-dimension). Both the wound healing test and transwell assay showed that MSC^CCR5^ cells responded robustly to hCCL5 stimulation, while only slight changes occurred for MSC^tdTomato^ cells (Fig. 7A-D). In addition, MSC^tdTomato^ and MSC^CCR5^ cells displayed relatively low migration without exogenous CCL5 treatment (Fig. 7B, D).

Given the dramatic upregulation of *Ccl5* in EAU retinas (Fig. 6C-E), we investigated whether CCR5-overexpression in MSC^CCR5^ cells would potentiate their chemotaxis towards EAU retinas. MSC^tdTomato^ and MSC^CCR5^ cells were transplanted into mice by caudal vein injection at 1 d.p.i. and the retinas were collected from transplanted EAU animals at 14 d.p.i. for tdTomato immunolabeling analysis (Fig. 7E). We found that tdTomato-positive cells were increased by more than 2.5-fold in retinas from MSC^CCR5^-transplanted EAU mice when compared with those from MSC^tdTomato^-transplanted animals (Fig. 7F, G). Therefore, these results suggest that CCR5 overexpression is able to enhance the CC chemokine-mediated chemotaxis of MSCs both in vitro and in vivo.

### Transplantation of MSC^CCR5^ cells ameliorates EAU

**Fig. 8:**
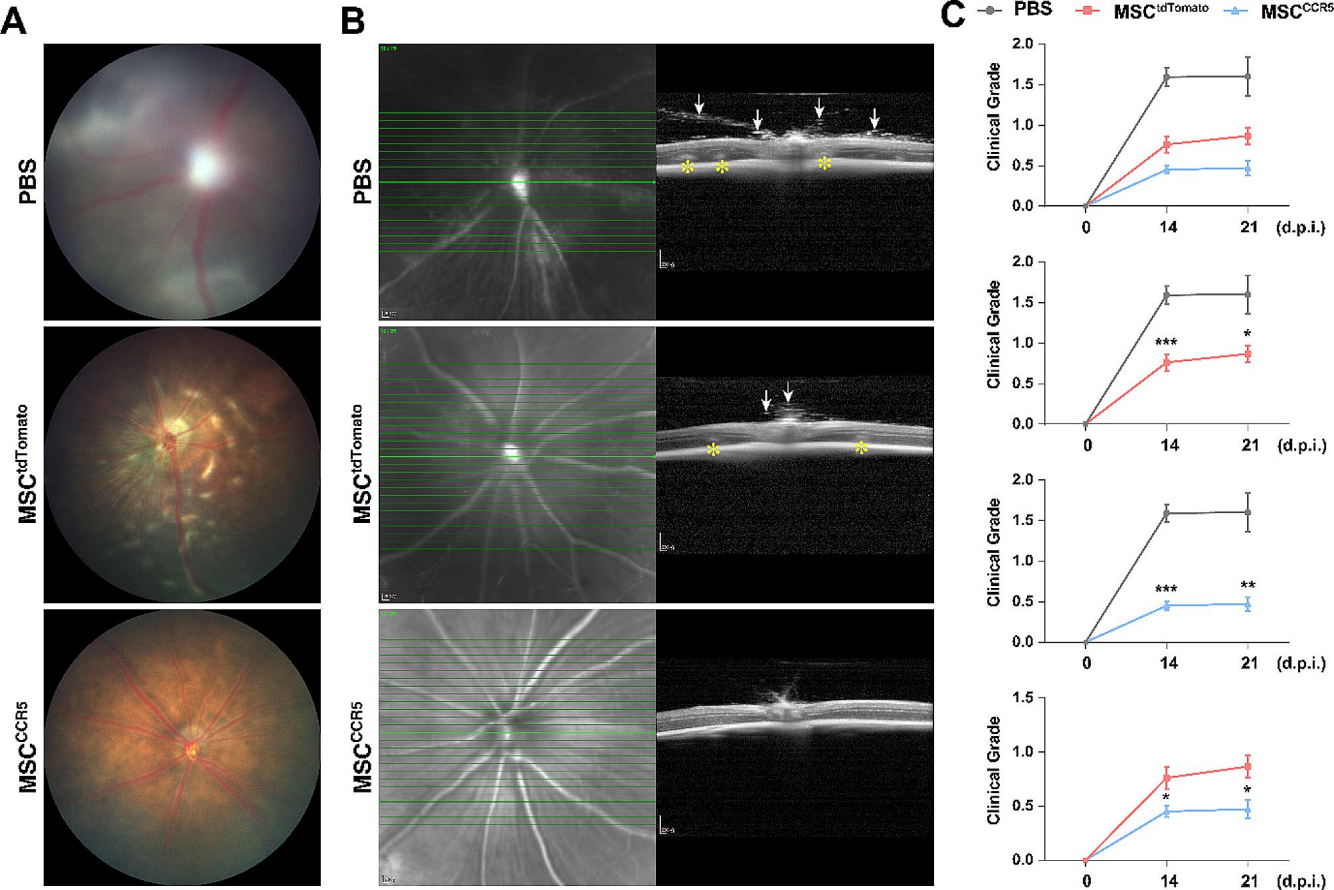
Transplantation of MSCs ameliorated EAU in vivo. (**A**) Representative fundus photographs of the eyes from EAU mice at 21 d.p.i., transplanted with MSC^tdTomato^ or MSC^CCR5^ cells, or administered with PBS. (**B**) Representative fundus photographs (left) and OCT images (right) of the eyes from EAU mice at 21 d.p.i., transplanted with MSC^tdTomato^ or MSC^CCR5^ cells, or administered with PBS. The arrows point to inflammatory infiltrating cells and the asterisks indicate retinal folds and detachment in EAU eyes. (**C**) Clinical grade scores of eyes in the PBS, MSC^tdTomato^ and MSC^CCR5^ groups at 0, 14 and 21 d.p.i. Data are presented as mean ± SEM (*n* = 12–32 eyes per group). *p < 0.05, **p < 0.001, ***p < 0.0001

Given that MSC^CCR5^ cells gain enhanced homing ability and that MSCs are known to ameliorate EAU [45, 46, 50, 51], we asked whether MSC^CCR5^ cells were better than regular MSCs in improving the clinical manifestations of EAU. At 14 and 21 d.p.i., by fundus imaging and OCT, we found that there were less structural damage and inflammatory infiltrates in the eyes of MSC-transplanted EAU mice compared to PBS-administered animals, and that MSC^CCR5^-transplanted EAU mice appeared to display even milder EAU symptoms than MSC^tdTomato^-transplanted ones (Fig. 8A, B; S6). Consistent with these observations, clinical grade scores at 14 and 21 d.p.i. were markedly lower for MSC-transplanted EAU mice compared with PBS-treated control animals (Fig. 8C). Moreover, MSC^CCR5^-transplanted EAU mice exhibited significantly lower clinical grade scores than those transplanted with MSC^tdTomato^ cells (Fig. 8C), indicating that CCR5-overexpressing MSCs have not only a stronger homing capacity but also better alleviating effect on EAU than regular MSCs.

### MSC^CCR5^ cells confer protection against microglia activation and T cell infiltration

**Fig. 9:**
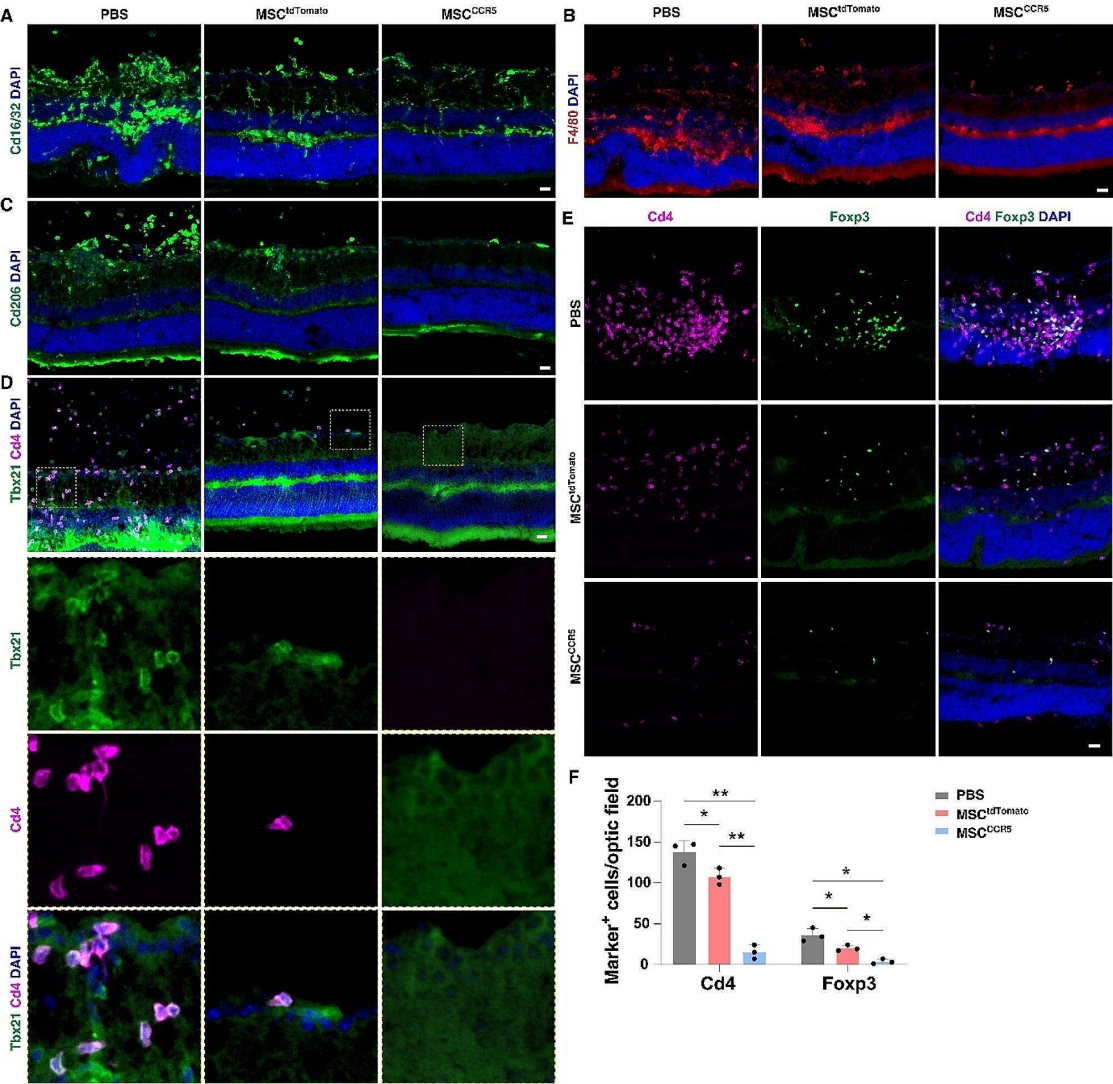
Transplantation of MSCs decreased microglia activation and T cell infiltration. (**A**-**E**) Retinal sections from EAU mice transplanted with MSC^tdTomato^ or MSC^CCR5^ cells, or administered with PBS were immunostained with antibodies against the indicated protein markers and counter-labeled with DAPI. Cd16/32 are markers for M1 microglia, F4/80 and Cd206 are markers for macrophages, and Tbx21, Foxp3 and Cd4 are markers for Th1 cells, Treg cells and T helper cells, respectively. (**F**) Quantification of Cd4-positive and Foxp3-positive cells in the indicated retinas. Data are presented as mean ± SEM. *p < 0.05, **p < 0.001 (*n* = 3 retinas per group). Scale bar: (**A**-**E**) 20 μm

To explore the cellular basis of MSC-mediated alleviation of EAU, we examined infiltrating immune cells in MSC^tdTomato^- and MSC^CCR5^-transplanted EAU retinas. Immunostaining showed that compared to control EAU retinas, MSC^tdTomato^-transplanted retinas contained significantly fewer pro-inflammatory M1 microglia positive for Cd16/32, and that the number of M1 microglia was reduced even more in MSC^CCR5^-transplanted retinas (Fig. 9A). Similarly, there were obviously fewer infiltrating F4/80^+^ or Cd206^+^ macrophages, Cd4^+^ T cells, Tbx21^+^ Th1 cells, and Foxp3^+^ Treg cells in MSC^tdTomato^-transplanted retinas, and their numbers were further decreased in MSC^CCR5^-transplanted retinas (Fig. 9B-F). These results together thus suggest that transplantation of CCR5-overexpressing MSCs at the time of immunization may block the progressive development of EAU, perhaps in part by preventing microglia activation and T cell infiltration.

### MSC^CCR5^ cells suppress activation of the Nlrp3 inflammasome

**Fig. 10:**
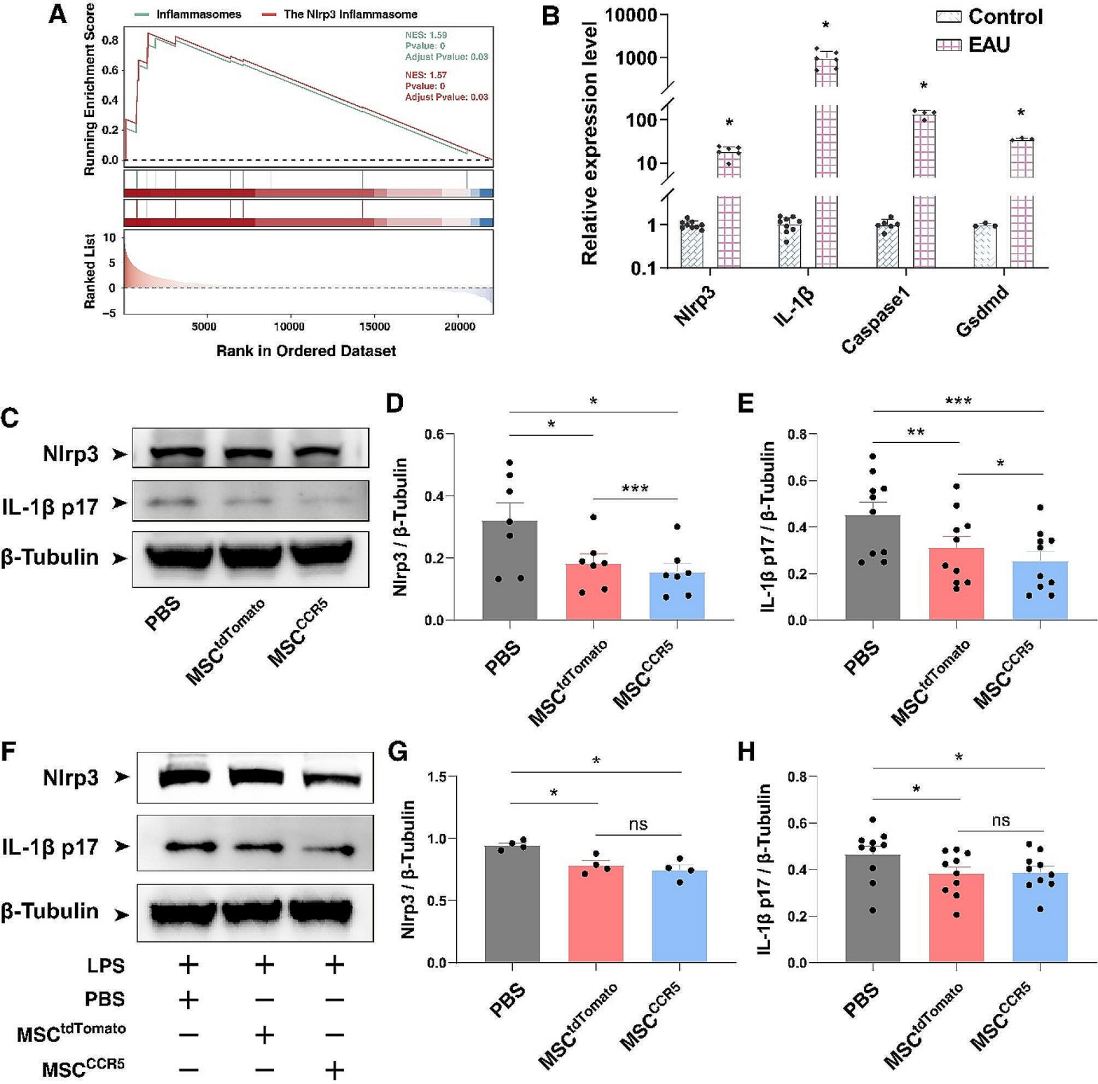
Transplantation of MSCs downregulated the expression of genes in the Nlrp3 inflammasome pathway. (**A**) GSEA enrichment analysis of the bulk RNA-seq data identified enriched gene sets associated with the inflammasomes and Nlrp3 inflammasome pathway. (**B**) qRT-PCR assay of the indicated genes involved in the Nlrp3 inflammasome pathway in control and EAU retinas at 14 d.p.i. Data are presented as mean ± SD (*n* = 3–9 retinas per group). *p < 0.0001. (**C**) At 21 d.p.i., Western blotting was performed for Nlrp3 and IL-1β p17 expressed in retinas of the PBS, MSC^tdTomato^ and MSC^CCR5^ groups. β-tubulin served as the internal protein control. (**D**, **E**) Quantification of relative protein expression levels of Nlrp3 and IL-1β p17 in retinas of the PBS, MSC^tdTomato^ and MSC^CCR5^ groups. Data are presented as mean ± SEM (*n* = 7–10 retinas per group). *p < 0.05, **p < 0.01, ***p < 0.001. (**F**) Western blotting analysis was performed for Nlrp3 and IL-1β p17 expressed in LPS-treated BV2 microglia cultured in the absence (PBS) or presence of MSC^tdTomato^ or MSC^CCR5^ cells. (**G**, **H**) Quantification of relative expression levels of Nlrp3 and IL-1β p17 in LPS-treated microglia cultured in the absence (PBS) or presence of MSC^tdTomato^ or MSC^CCR5^ cells. Data are presented as mean ± SEM (*n* = 4–10 individual samples per group). *p < 0.05, ns, no significance

Previous studies have shown that the primary function of retinal microglia in autoimmune uveitis is to initiate the disease, and without microglia, no local inflammation would develop; moreover, microglia produce cytokines such as IL-1β and IL-18 by the activation of inflammasomes [52–57]. Additionally, the activation of the Nlrp3 inflammasome and the maturation of IL-1β have been reported in the autoinflammatory uveitis, such as Behçet’s disease [58]. We therefore investigated whether EAU would activate inflammasomes in the retina. By GSEA enrichment analysis of the bulk RNA-seq data, we found that the DEGs were indeed significantly enriched for genes associated with the inflammasomes and Nlrp3 inflammasome pathway (Fig. 10A). And in the scRNA-seq data, the Nlrp3 inflammasome genes were also seen to be upregulated mainly in microglia, monocytes/macrophages, and T/NK cells in EAU retinas (Figure S7A). At 14 d.p.i., qRT-PCR assay confirmed that the genes involved in the Nlrp3 inflammasome pathway (e.g. *Nlrp3, IL-1β, Caspase1, and Gsdmd*) were significantly upregulated in EAU retinas compared to controls (Fig. 10B), indicating that EAU results in Nlrp3 inflammasome activation.

Given the relieving effect of MSCs on EAU, we next tested whether they would also inhibit Nlrp3 inflammasome activation. Western blot analysis showed significant downregulation of inflammasome-related proteins Nlrp3 and IL-1β in MSC-transplanted retinas compared to controls, and their expression levels were the lowest in MSC^CCR5^-transplanted retinas (Fig. 10C-E). Consistent with the in vivo results, lipopolysaccharide (LPS)-treated BV2 microglia co-cultured with MSCs exhibited reduced expression of Nlrp3 and IL-1β (Fig. 10F-H), suggesting that MSCs may also ameliorate EAU by inhibiting Nlrp3 inflammasome activation in microglia. Similarly, we were able to show that CCR5-overexpressing MSCs inhibited NLRP3 inflammasome activation in THP-1 monocytes-derived macrophages (Figure S7B).

## Discussion

In this study, we applied scRNA-seq and bulk RNA-seq to characterize the classical EAU induced by IRBP peptide 651–670 in C57BL/6 mice. Based on the patterns of gene expression in resident retinal cells and infiltrating immune cells, and the results of immunostaining and qRT-PCR analyses, we observed/inferred that: (1) EAU caused a decrease in expression levels of a set of retinal neuron marker genes and progressive degeneration of all retinal neuron types; (2) Müller glia might be activated and function as non-professional APCs in EAU retinas; (3) Th1 cells were the predominant class of T helper cells in the retinas of mice with IRBP peptide 651-670-induced EAU; and (4) Cytokines were widely involved in EAU and CCL5 was the most expressed CC chemokine ligand in EAU retinas. Based on these findings, we proposed and tested a strategy to ameliorate EAU by transplantation of MSC^CCR5^ cells overexpressing human CCR5. Compared to regular MSCs, MSC^CCR5^ cells increased targeted migration toward CCL5 both in vitro and in vivo, better protected against microglia activation and T cell infiltration, and exhibited a better therapeutic effect on EAU. Therefore, CCR5-overexpression in MSCs enhances their homing capacity and improves therapeutic outcomes, thereby providing an appealing MSC-based therapy option.

When autoimmune uveitis encompassed the retina, it was often associated with autoreactivity to retinal antigens such as the S-antigen (Arrestin) and IRBP, and this was referred to as uveoretinitis [16, 24, 59–73]. S-antigen was highly efficient in the induction of EAU in many different animal species including rats, rabbits, guinea pigs, and primates [74], and autoimmunity to Arrestin was crucial for the pathology of uveitis [75]. Our experimental results of scRNA-seq, bulk RNA-seq, and immunolabeling showed that in the IRBP-induced EAU retinas, the expression of molecular markers of all 6 neuron types was downregulated and these neuron types underwent progressive degeneration (Fig. 2). As expected, the number of cones decreased significantly with EAU disease progression, but scRNA-seq analysis revealed that EAU appeared to affect the number of rods less than cones although IRBP mRNA was detected in both cones and rods [76, 77] (Fig. 2). This phenomenon may be explained by a theory called “epitope spreading”: autoimmunity starts with an immune response to a single antigen, and subsequently extends to other proteins in the same tissue, or to different amino acid sequences within the same molecule (epitope) [75, 78]. Based on this theory, we have hypothesized that when EAU is induced by IRBP peptide 651–670, the immune response may be first directed to IRBP and then extended to Arrestin so that the cones are attacked more by immune cells than rods.

Apart from the impact on retinal neurons, EAU also caused changes in the state of Müller glia, the major glial component of the retina. Under physiological conditions, Müller glia are thought to contribute to ocular immune privilege by inhibiting the proliferation and activation of lymphocytes [31]. They can be induced to generate neurons [79–85], express the MHC class II proteins, and stimulate the proliferation of T cells as APCs in an MHC class II-dependent manner [86, 87]. In the present work, EAU did not appear to affect the number of Müller glia but upregulated the expression of their marker genes (Fig. 2). Interestingly, *Gfap*, an indicator of the activated state of Müller glia, was upregulated in only one Müller glia cluster present mainly in EAU retinas (Fig. 3). Moreover, MHC class II proteins were found to be colocalized with Müller glial markers Sox9 (in cell bodies) and glutamine synthetase (in radial processes) (Fig. 3). Thus, consistent with a previous study [16], Müller glia may also act as non-professional APCs in the context of EAU. In addition, our single-cell sequencing data and immunostaining showed that some VECs, astrocytes, microglia and monocytes/macrophages expressed the MHC II proteins (Figure S3E). Hence, further study is needed to elucidate the proportion of Müller cells acting as non-professional APCs compared to other non-professional APCs, and to determine the relative contribution of Müller glia compared to other APCs in the uveoretinitis disease process. A reliable method to distinguish astrocytes and Müller glia deserves further exploration because the expression profile of Müller glia changed in EAU.

The accumulation of leukocytes in target organs is a sign of autoimmunity [88]. The absence or near-absence of lymphoid cells in wild-type (WT) retinas may be a feature of ocular immune privilege [89]. Activated T cells specific to retinal antigens mediate EAU in animals, and T cells are also central to the pathogenesis of human uveitis [9, 90, 91]. Our results showed that in WT control retinas, a small number of microglia and monocyte-lineage cells were present, but T and NK cells were nearly undetectable; but in EAU retinas, there were many more nonresident immune cells, and there was a preponderance of T and NK cells and monocyte-lineage cells (Figs. 2 and 4). By scRNA-seq analysis and immunostaining of EAU retinas, we observed aggregates of multiple types of T cells (Fig. 4). Similarly, a recent study by Peng et al. showed that multiple types of T cells infiltrated into the EAU retina, and that the ratio of Th1 cells in the retina was highest on 14 d.p.i. and 21 d.p.i. [92]. Moreover, it has been suggested that Th1 cells are mainly involved in the development of acute uveitis, whereas Th17 cells play a role in the late or chronic phase of uveitis [93]. An increase in Th17 cells was observed in the retina over time [92], suggesting that the major pathogenic T cells may change over the EAU disease process. Future studies targeting later or more severely graded EAU retinas would help to understand the dynamics throughout the course of the disease. The predominance of Th1 cells as effector T cells in the classical EAU retinas is inconsistent with previous reports that complete Freund’s adjuvant (CFA) promoted a Th17-dominant response [12, 13]. Conceivably, there are at least two reasons why the differences exist: (1) Chen et al. induced EAU in the B10.RIII mice instead of C57BL/6 mice [12]; and (2) it was IRBP peptide 161–180 rather than IRBP peptide 651–670 that was used in their study [12]. Previous studies have shown that autoimmunity to the retina can be either Th17- or Th1-driven, depending on the conditions of initial exposure to antigens [13, 89]. And it is known that uveitis is clinically heterogeneous, even though patients may respond to the same retinal antigen(s) [9]. Moreover, previous observations from EAU and other models of autoimmune diseases have demonstrated that the immune systems respond to different fragments of a given molecule, and that cell-mediated and humoral responses are invariably directed against different parts of the molecule [94]. Our data therefore reinforce the notion that no single animal model can reproduce the full spectrum of features of the uveoretinitis disease.

Previous studies have revealed the importance of chemokines in the pathogenesis of uveitis [51, 95, 96], and chemokines may be involved in the initiation and/or amplification steps in the pathogenicity of uveitis [18, 96]. In addition, chemokines are powerful chemoattractants for T cells [97]. Our scRNA-seq and bulk RNA-seq data revealed that a number of cytokines were enriched in EAU retinas and that the CC chemokine family exhibited a more significant change between EAU and control retinas than other chemokine families. We examined the expression of about a dozen of CC chemokines in EAU retinas by qRT-PCR assay and found that *Ccl5* displayed the most significant change postimmunization. It has been reported that CCR5, a receptor of CCL5, plays an important role in the differentiation of Th1 cells [38–41, 98]. In this context, the dramatic upregulation of CCL5 may explain why Th1 cells are the predominant T helper cells in EAU retinas. Nevertheless, *CCR5*-deficient mice develop EAU with a degree of severity comparable to that in wild-type mice because the reduced T-cell infiltration caused by *CCR5* deletion was compensated by augmented granulocyte infiltration [19]. *CCR5* knockout may cause a significant shift in the gene expression profile of immune cells that ultimately results in changes in their chemotaxis. As such, *CCR5* inactivation may not be an appropriate strategy for treating EAU. In addition, it remains unclear whether CCL5 plays a role in the initiation or amplification of EAU, due to the fact that we did not detect *Ccl5* mRNA at different time points during the progression of EAU. Related to this, the relative contribution of Th1 and Th17 cells at different stages of EAU needs to be further explored because a previous study suggested that Th17 cells were responsible for ocular inflammation in the early phase of EAU, whereas Th1 cell activation was correlated with the late stage and regression of inflammation [99].

In recent years, MSC transplantation has become a promising therapy option for the treatment of immunological and inflammatory disorders, including graft-versus-host disease [100–102], spinal cord injury [103–105], stroke [23, 106, 107], and of course EAU [45, 46, 50, 51, 108]. In MSC therapy, it would be ideal that most of the infused cells are recruited to the target lesions or tissues [107, 109, 110]. However, it is widely believed that exogenously administered cells are rapidly cleared by the host and do not engraft for long periods [43, 111]. Although in some preclinical and clinical trials, MSCs are directly injected into the target lesions or tissue [112–114], local administration may cause potential invasive damage. Thus, it is imperative to develop new approaches to enhance infused MSCs administered by a relatively safe route, such as intravenous injection, with improved homing capacity to the target lesions. Additionally, amelioration of EAU has been achieved by inhibition of migration and recruitment of inflammatory cells into the eye by targeting adhesion molecules and/or chemokines and chemokine receptors [9, 18, 115]. Considering that: (1) the expression level of CCL5 was the highest among CC chemokines in EAU retinas (Fig. 6); (2) the number of CD4^+^ T cells and Th1 cells was increased by EAU immunization (Fig. 4); and (3) isolated MSCs were found to gradually lose their homing capacity and exhibit functional heterogeneity [21–23, 49], we generated functional and homogeneous hiPSC-derived MSCs and overexpressed hCCR5 in MSCs via lentiviruses. As expected, an increased number of MSC^CCR5^ cells was observed in EAU retinas, exerting superior protection compared to regular MSCs (Figs. 7, 8, 9 and 10).

Previous work has demonstrated that microglia initiate neuroinflammation in ocular autoimmunity and mediate the entry of autoreactive immune cells including T cells into the retina [53]. We found that after MSC^CCR5^ treatment, the structure of the retina was more intact (Fig. 8) and the number of Cd16/32^+^ M1 microglia, infiltrating F4/80^+^ or Cd206^+^ macrophages, Cd4^+^ T cells, Tbx21^+^ Th1 cells, and Foxp3^+^ Treg cells in the EAU retina was drastically reduced (Fig. 9). Nevertheless, Treg cells were believed to suppress Th1 immune responses in uveitis [116], but they obviously decreased in EAU retinas after MSC transplantation. A possible explanation for this is that cell-based therapies have the potential to restore ocular immune privileges, preventing all types of lymphocytes from infiltrating into the retina.

In EAU retinas, the inflammasome pathway was enriched and Nlrp3 inflammasome-related genes including *Nlrp3, Caspase1, Gsdmd*, and *Il1b* (IL-1β) were upregulated at RNA levels (Fig. 10). MSC^CCR5^ treatment significantly downregulated the expression of Nlrp3 and IL-1β. Similarly, their downregulation was confirmed in LPS-treated BV2 microglia after co-culture with MSCs (Fig. 10). Taken together, we have revealed a possible mechanism underlying the beneficial effects of MSC^CCR5^ therapy, which may rely on inhibiting the activation of microglia and inflammasomes, leading to diminished release of inflammatory factors and thereby reducing the infiltration of autoreactive immune cells such as T cells into the retina.

## Conclusions

In this extensive study employing scRNA-seq and bulk RNA-seq, our investigation delved into the complexities of EAU induced by the IRBP peptide 651–670 in C57BL/6 mice, and uncovered a multifaceted impact of EAU on retinal components, revealing a decrease in the expression of retinal neuron marker genes and progressive degeneration of various retinal neuron types. Additionally, the study shed light on the intriguing role of Müller glia, which, under EAU conditions, displayed activation and potential function as non-professional APCs. The immune cell landscape in EAU retinas unveiled a prevalence of Th1 cells, contrary to previous reports, underscoring the heterogeneity of uveitis. Cytokine involvement, particularly the pronounced expression of CCL5, hinted at its potential role in orchestrating the immune response. We extended this study by proposing and testing a therapeutic strategy involving the transplantation of CCR5-overexpressing MSCs, which showed enhanced homing capacity and promising outcomes in preventing EAU. These specialized cells provided improved protection against microglia activation and T cell infiltration. Notably, MSC^CCR5^ treatment downregulated Nlrp3 inflammasome-related genes, suggesting a mechanism for reducing inflammation and preventing autoreactive immune cell infiltration. This work not only contributes valuable insights into the molecular characteristics of EAU but also opens avenues for innovative MSC-based therapies. Nonetheless, there is still a long way to go for clinical application. More details about MSC transplantation, such as the methods for modifying MSCs, the time point of transplantation, the number of transplanted cells, and the frequency of transplantation, should be evaluated comprehensively.

## Materials and methods

### Animals

All experiments on mice were performed according to the IACUC (Institutional Animal Care and Use Committee) standards and approved by Sun Yat-sen University and Zhongshan Ophthalmic Center. The C57BL/6 mice were purchased from the Vital River Laboratories (Beijing). Mice were maintained in pathogen-free facilities under standard housing conditions with continuous access to food and water. All experiments were carried out in adult mice from 8 to 11 weeks of age. The contribution of gender was not considered in this paper. To control for sex effects, only female mice were studied.

### Experimental autoimmune uveitis induction and assessment

EAU was induced in mice using a protocol described previously [24] with minor modifications. Briefly, anesthetized 8-week-old C57BL/6 mice (50 mg/kg sodium pentobarbital, Tocris, 4579) were immunized with 200 µg human IRBP peptide 651–670 (LAQGAYRTAVDLESLASQLT, Shanghai Hanhong, CSP0725) which was emulsified in Complete Freund’s Adjuvant (1:1 vol/vol, CFA, Sigma, F58881) containing an additional 3.5 mg/mL *Mycobacterium tuberculosis* H37Ra (BD, 231,141), by injection with a 30G needle into the two thighs (50 µg each) and the tail base (100 µg). Concurrent with immunization, 1 µg of pertussis toxin (PTX, List Biological, 180) was injected intraperitoneally (recorded as day 0), followed by another injection of 1 µg PTX at 2 d.p.i. Every 7 days postimmunization, the eyes of these animals were inspected and graded for disease induction based on the criteria described previously (Table S1) [24, 25]. Retinas were collected at 14 d.p.i. or 21 d.p.i. for desired experiments.

### Fundus imaging, fluorescein fundus angiography (FFA), and optical coherence tomography (OCT) imaging

For all clinical examinations [Fundus imaging, FFA, and OCT], mice were anesthetized with sodium pentobarbital (50 mg/kg, Tocris, 4579). Local ocular surface anesthesia was achieved by 0.5% dicaine hydrochloride eye drops (Zhongshan Ophthalmic Center). The pupils were dilated using 0.5% tropicamide and 0.5% phenylephrine hydrochloride (Shenyang Sinqi Pharmaceutical Co., Ltd.). Artificial tears (Zhongshan Ophthalmic Center) or 1% hypromellose eye drops (Zhongshan Ophthalmic Center) were used to maintain corneal moisture and clarity. The fundus was imaged using the Phoenix Micron IV in vivo retinal imaging microscope. For FFA, 150 mg/kg fluorescein sodium (Alcon) was injected and the fundus images were captured in 5 min. OCT images were obtained using the Heidelberg Engineering Spectralis HRA + OCT.

### Single-cell RNA sequencing

scRNA-seq analysis was carried out as previously described with modification [52, 117]. In brief, retinas were quickly dissected in Dulbecco’s phosphate-buffered saline (DPBS, Hyclone, SH30028.02) and digested using papain (Worthington Biochemicals, LS003126) with DNase I (Roche, 10,104,159,001) at 37 °C for 5 min. Then isometric amount of DPBS containing 10% fetal bovine serum (FBS, Genial, G11-70500) was added and retinas were triturated by soft pipetting. The dissociated cells were filtered using a 40 μm cell strainer (Falcon, 352,340). Filtered cells were centrifuged and resuspended in 0.4% bovine serum albumin (BSA, Sigma, B2064) in DPBS. Cell viability was determined by Cellometer (Nexcelom) and only cell suspension with a viability of more than 85% was subjected to scRNA-seq analysis. Single-cell libraries were constructed from the resuspended cells according to the manufacturer’s instructions using the Chromium Single Cell 3′ Library & Gel Bead Kit v3.1 (10X Genomics, 1,000,121), and sequenced on the Illumina 10X platform. Quality checks were performed using the FastQC software (https://www.bioinformatics.babraham.ac.uk/projects/fastqc/). scRNA-seq data were pre-processed using the Cell Ranger pipeline (v6.0.1; 10X Genomics) with default settings and mm10 as the reference genome. Further analysis was performed using Python and R packages: Scrublet [118], Seurat [28, 29], and CellChat [36]. scRNA-seq data reported in this paper were deposited in the NCBI Sequence Read Archive (SRA) database under accession numbers SRR25528276 and SRR25528277.

### Bulk RNA sequencing

Bulk RNA-seq analysis was performed as previously described [119] with modification. Total RNA was extracted using NucleoZOL reagent (MACHEREY-NAGEL, 740404.200) following the manufacturer’s procedure. The quantity and purity of total RNA were analyzed with Bioanalyzer 2100 and RNA 6000 Nano LabChip Kit (Agilent, 5067 − 1511), and only high-quality RNA samples with RIN number > 7.0 were used to construct sequencing libraries. The average insert size for the final cDNA libraries was 300 ± 50 bp. We performed the 2 × 150 bp paired-end sequencing (PE150) on an Illumina Novaseq™ 6000 (LC-Bio Technology CO., Ltd.) following the vendor’s recommended protocol. We aligned reads of all samples to the mouse reference genome (mm10) using the HISAT2 software (https://daehwankimLab.github.io/hisat2/). Hierarchical cluster and scatter plot analyses of gene expression levels were performed using the R software (http://cran.r-project.org). Gene ontology (GO) analysis, Kyoto Encyclopedia of Genes and Genomes (KEGG) analysis, and Gene Set Enrichment Analysis (GSEA) were carried out using the clusterProfiler [120] package with p value < 0.05. P value < 0.05 and log2FoldChange ≥ 1.5 were set as the significant threshold in the DEG (differentially expressed gene) analysis with the DESeq2 package [121]. |NES| > 1 and p.adjust < 0.05 were set as the significant threshold in GSEA. RNA-seq data reported in this paper were deposited in the NCBI SRA database under accession numbers SRR25528270-SRR25528275.

### Quantitative real-time PCR (qRT-PCR)

Retinas of WT control and EAU mice were dissected out of the eyeball and harvested. Total RNA was isolated using the NucleoZOL reagent. RNA (1 µg) from each sample of different groups was converted to cDNA using the HiScript II Q RT SuperMix for qPCR (Vazyme Biotech, R223-01). qRT-PCR was performed using the Kapa SYBR fast qPCR master mix (Kapa, KK4601) and qTOWER3 G Real-Time PCR system (Analytikjena). The data were analyzed using the 2^−ΔΔct^ calculation method. All data were tested for significance using the two-sample Student’s t-test. The primer sequences used for qRT-PCR are listed in Table S4.

### iPSC culture and MSC induction

We utilized one human induced pluripotent stem cell (hiPSC) line that was previously established in our lab [122]. The cells were cultured on Matrigel (Corning, 354,277)-coated plates in mTeSR1 medium (Stemcell Technologies, 05445). The medium was changed daily and the cells were passaged every 4–5 days using Accutase (Life Technologies, AT-104).

The hiPSCs were differentiated into MSCs as previously described [49]. First, to differentiate hiPSCs into neuromesodermal progenitors (NMPs), these cells (1–2 × 10^5^ cells/cm^2^) were seeded on Matrigel-coated plates and cultured in mTeSR1 medium containing 10 µM Y27632 (Sigma, Y0503) for 24 h. Neuromesoderm differentiation was initiated by culturing cells in E6 medium (Stemcell Technologies, 05946) supplemented with 20 ng/mL bFGF (Peprotech, 100-18B), 3 ng/mL TGFβ1 (Peprotech, 100 − 21), and 10 µM Chir99021 (TargetMol, T2310) for 5 days. Next, for MSC differentiation, the NMPs were cultured for another 3 weeks in animal component-free and serum-free medium (MesenCult™-ACF Plus Medium; Stemcell Technologies, 05448). The resulting cells were maintained in MesenCult™-ACF Plus medium (Fig. S5).

The phenotype and multipotency of hiPSC-derived MSCs were assessed by flow cytometry analysis and by their ability to differentiate into mesenchymal-lineage cells (osteoblasts, adipocytes, and chondrocytes). For osteogenic differentiation, cells were fixed and incubated with Alizarin Red S (Sigma, A5533) for 30 min for the detection of calcium deposits. For adipogenic differentiation, cells were fixed and incubated with Oil Red O (Sigma, O0625) for 20 min for the detection of lipid droplets. For chondrogenic differentiation, cells were fixed and incubated with 0.1% toluidine blue (Sigma, 89,640) for 10–15 min. Labeled cells were captured with a microscope (Fig. S5).

### Flow cytometry

MSCs were dissociated into single-cell suspension using Accutase, washed twice in 0.2% cold BSA, and precipitated by centrifugation (500 g at 4 °C). The cell pellet was resuspended in 0.2% cold BSA, and incubated for 30 min with the appropriate antibodies (Table S5) on ice protected from light. The cells were then washed twice and precipitated to remove unbound antibodies. The cell pellet was resuspended in 300 µL 0.2% BSA in a 5-mL round bottom tube (Falcon, 352,235) for flow cytometric analysis (Fig. S5). All flow cytometric analysis was conducted with the BD flow cytometer (LSRFortessa™ X-20) and the data were analyzed using the BD FlowJo software (FlowJo, Ashland, OR, USA).

### Construction of viral plasmids and lentivirus preparation and infection

The lentiviral expression vectors used in the present study were designated as pLV/puro-EF1α-CCR5-T2A-tdTomato and pLV/puro-EF1α-tdTomato, which were constructed using the gateway method (Fig. S5).

Lentivirus particles were harvested from 293T cells as described [52]. In brief, the viral constructs and package plasmids Pspax2 (Addgene #12,260) and PMD2.G (Addgene #12,259) were transfected into HEK293T cells using the Hieff Trans® Liposomal Transfection Reagent (Yeasen, 40802ES08) according to the manufacturer’s instruction. After 48 h, the culture supernatants were harvested and filtered through a 0.45 μm sterile filter (Millipore, SLHV033RB), then ultra-centrifuged at 20,000 g (rotor SW32Ti, Beckman) for 1.5 h at 4℃ to precipitate the lentiviruses.

MSCs, at 70 ~ 80% confluence in a well of a 6-well plate (Corning, 3516), were infected with the pLV/puro-EF1a-CCR5-T2A-tdTomato or pLV/puro-EF1a-tdTomato viruses in 2 mL fresh MesenCult™-ACF Plus medium in the presence of 10 µg/mL polybrene (Sigma, TR-1003-G). After 16 h, the virus and medium mixture was replaced with fresh MesenCult™-ACF Plus medium.

### Fluorescence activated cell sorting (FACS)

Three days after lentivirus infection, MSCs were dissociated using Accutase. After enzyme inactivation, the cell suspension was centrifuged at 186 g for 5 min. The precipitated cells were resuspended in DPBS containing 2% FBS and 1 mM EDTA, and passed through a 40 μm cell strainer (Falcon, 352,340) into a 5 mL round bottom tube (Falcon, 352,003). MSC^CCR5^ and MSC^tdTomato^ cells were purified by FACS (BD, FACSAriaIII), gated for a high level of tdToamto expression. The sorted MSC^CCR5^ and MSC^tdTomato^ cells (Fig. S5F) were used for the wound healing test, transwell assay, transplantation, and co-culture.

### HEK293T cell, BV2 microglia and THP-1 monocyte culture

The HEK293T (293T) cells (ATCC, CRL-3216) and THP-1 monocytes (ATCC, TIB-202) were purchased from ATCC, and the BV2 microglia were from iCell Bioscience (iCell-m011). These cells were tested for mycoplasma contamination before experiments and they were all negative. The 293T cells and BV2 microglia were expanded in the Dulbecco’s modified Eagle’s medium (DMEM, Hyclone, SH30243.01B) supplemented with 10% FBS (Gibco, 10270-106), 1X Penicillin-Streptomycin (Gibco, 15,140,122), and 1X MEM non-essential amino acids (Gibco, 11,140,050). The THP-1 monocytes were cultured in RPMI 1640 medium (ThermoFisher, 11,875,093) supplemented with 10% FBS, 1X Penicillin-Streptomycin, and 0.05 mM β-Mercaptoethanol (Sigma, M3148). The cell incubator provided an environment of 5% CO_2_ and 37℃.

### Wound healing test and transwell assay

The migrative capacity of MSCs was evaluated using a 3-well silicone insert (for 2-dimensional migration, Ibidi, 80,369) and an 8 μm-pore transwell chamber system (for 3-dimensional migration, Corning, 3422).

In wound healing test, MSCs were seeded into the 3-well culture-insert and cultured until an optically confluent cell monolayer was formed. Cell proliferation was then suppressed by treating the cells with the proliferation inhibitor, mitomycin C (1 µg/mL, Selleck, S8146) for 1 h. The culture-insert was removed to create gaps, followed by replacement of the old medium with fresh one with or without 100 ng/mL human CCL5 (R&D system, 278-RN). Gap closure was monitored by taking pictures at different time points (0, 3, 6, 12, 24 h) under an inverted optical microscope.

In the transwell assay, MSCs were seeded onto the upper chamber (2.0 × 10^5^ per well) placed in a 24-well plate (Corning, 3524), while the lower chamber was loaded with recombinant human CCL5 (100 ng/mL). After 4 h of co-culture, we swabbed the MSCs remaining on the upper surface of the membrane and stained the filter with 0.1% crystal violet (Solarbio, G1064). MSCs that migrated to the lower surface of the membrane were counted under an inverted optical microscope.

### Cell transplantation

Our experimental design consisted of 3 groups: Group 1, PBS alone (0.1 mL without MSC administration); Group 2, MSC^tdTomato^ transplantation group (2.0 × 10^6^ cells/mouse); and Group 3, MSC^CCR5^ transplantation group (2.0 × 10^6^ cells/mouse). Cells were suspended in 0.1 mL of the DPBS and all injections were performed at 1 d.p.i. via the caudal vein (Fig. 7E). After 13 days (14 d.p.i.) or 20 days (21 d.p.i.) of transplantation, mice were euthanized and retinas were collected for subsequent experiments.

### Hematoxylin-eosin (HE) staining

Eyeballs were fixed overnight in the FAS Eyeball Fixator (Servicebio, D60708) and sectioned in paraffin at 15 μm, followed by HE staining. Sectioning and staining were carried out by Servicebio.

### Co-culture and LPS treatment

The BV2 microglia were indirectly co-cultured with MSCs using the 0.4 μm-pore transwell chamber system (Corning, 3412) as previously described [123]. They were seeded in a 6-well plate. Another 6-well plate was prepared, in which the transwell inserts were carefully placed. 500 µL MSC^CCR5^ or MSC^tdTomato^ cell suspension (1 × 10^6^ cells/mL) was seeded onto the transwell insert. Isometric amount of DPBS was added into the insert as the control group. The cells were cultured at 37℃ and 5% CO_2_ for ~ 24 h until BV2 microglia reached about 95% confluence. Subsequently, the culture medium was replaced with the medium containing lipopolysaccharide (LPS, 100 ng/mL, Sigma, L4391). Meanwhile, the MSC medium was also replaced with the BV2 microglia medium containing LPS (100 ng/mL). The inserts were then placed into the wells of the 6-well plate seeded with BV2 microglia. After 4 h of culture, the BV2 microglia were washed twice with DPBS and processed for Western blotting analysis.

The THP-1 monocytes (5 × 10^5^ cells/well) were seeded on 6-well plates and treated with 150 nM phorbol 12-myristate 13-acetate (PMA, Sigma, P1585) for 24 h for them to transform into adherent macrophages. Meanwhile, the MSC^CCR5^ and MSC^tdTomato^ cells were prepared as described above. They were then indirectly co-cultured with the THP-1 monocytes-derived macrophages and the culture medium was replaced with the fresh medium containing 100 ng/mL LPS. After 4 h of co-culture, the macrophages were collected for qPCR analysis.

### Western blotting

Total protein was isolated from retinas at 21 d.p.i. or BV2 microglia using the RIPA buffer (Beyotime, P0013B) supplemented with PMSF (phenylmethanesulfonyl) (Beyotime, ST505) and quantified using the BCA kit (Beyotime, P0010). Samples were loaded on gradient gels (GenScript, M00657) for protein separation. The expression levels of target proteins were quantified by ImageJ and normalized to β-tubulin in the same sample. Primary and secondary antibodies for Western blotting are listed in Tables S6 and S7, respectively.

## Immunostaining

Immunofluorescence staining of retinal sections was carried out as previously described [80, 124]. Briefly, for section labeling, retinas were fixed in 4% paraformaldehyde in PBS for 30 min at 4 °C and sectioned at 15 μm. Sample sections were washed 3 times with 0.4% Triton X-100 in PBS (PBST) for 5 min each before being incubated in 5% normal donkey serum in PBST for 1 h at room temperature (RT). The primary antibodies (Table S6) in 1% normal donkey serum in PBST were added for overnight incubation at 4 °C. After washing with PBST, the sections were incubated with secondary antibodies (Table S7) and DAPI in PBST for 1 h at RT.

Immunohistochemical staining was performed as previously described [125, 126]. Briefly, eyeballs were fixed overnight in FAS Eyeball Fixator and sectioned in paraffin at 15 μm. Then, sections were deparaffinized, subjected to antigen retrieval, incubated in 3% hydrogen peroxide to eliminate endogenous peroxidase activity, blocked in solution containing 5% normal donkey serum, 3% BSA, and 0.2% Triton X-100, incubated with the primary antibodies (Table S6) overnight at 4 °C and the secondary antibodies (Table S7) for 1 h at RT. Finally, sections were sequentially stained with the AEC substrate (Solarbio, A2010) and hematoxylin (Servicebio, G1004).

Images were captured by a laser scanning confocal microscope (Carl Zeiss, LSM700).

### Statistical analysis

For quantification of marker-positive cells in retinal sections, optic field represents the area acquired by laser confocal microscopy at the same magnification. All experiments have at least three biological replicates. At least one retina was counted per mouse and three sections were counted per retina. Two optic fields were counted per section. Statistical analysis was performed using GraphPad Prism 8.0, R, and Python. The results are expressed as mean ± SD or mean ± SEM for experiments conducted at least in triplicates. Student’s t test was used to assess differences between two groups, and one-way ANOVA with Bonferroni’s correction was used to assess differences among three groups. A value of *p* < 0.05 is considered statistically significant.

### Electronic supplementary material

Below is the link to the electronic supplementary material.

Supplementary Material 1

Supplementary Material 2

## Data Availability

The data are available from the corresponding author on reasonable request. The scRNA-seq data are deposited in the NCBI SRA database under accession numbers SRR25528276 and SRR25528277 and bulk RNA-seq data are also deposited in the NCBI SRA database under accession numbers SRR25528270-SRR25528275.

## Abbreviations

AC: amacrine cell
APCs: antigen presenting cells
BC: bipolar cell
BRB: blood-retinal barrier
BSA: bovine serum albumin
CFA: complete Freund’s adjuvant
d.p.i.: days postimmunization
DEGs: differentially expressed genes
DMEM: Dulbecco’s modified Eagle’s medium
DPBS: Dulbecco’s phosphate-buffered saline
EAU: experimental autoimmune uveitis
FACS: fluorescence-activated cell sorting
FBS: fetal bovine serum
FFA: fluorescein fundus angiography
GCL: ganglion cell layer
GO: Gene Ontology
GS: glutamine synthetase
GSEA: Gene Set Enrichment Analysis
HC: horizontal cell
INL: inner nuclear layer
iPSC: induced pluripotent stem cell
IRBP: interphotoreceptor retinoid-binding protein
KEGG: Kyoto Encyclopedia of Genes and Genomes
MG: Müller glia
MHC: major histocompatibility complex
MSCs: mesenchymal stem cells
NK: natural killer cell
NMP: neuromesodermal progenitor
OCT: optical coherence tomography
ONL: outer nuclear layer
PBST: Triton X-100 in PBS
PTX: pertussis toxin
PMA: phorbol 12-myristate 13-acetate
RGC: retinal ganglion cell
RNA-seq: RNA sequencing
RT: room temperature
S: inner and outer segment
scRNA-seq: single-cell RNA sequencing
VEC: vascular endothelial cell
