# Supplementary material for: CCR5-overexpressing mesenchymal stem cells protect against experimental autoimmune uveitis: insights from single-cell transcriptome analysis
Source: J Neuroinflammation. 2024 May 27;21:136. doi: 10.1186/s12974-024-03134-3 (PMC11131209; doi:10.1186/s12974-024-03134-3)
Supplement: Supplementary file 1 — Supplementary Material 1 [file 12974_2024_3134_MOESM1_ESM.docx]

**

**

**Uncropped Blots images.** (A-C) Blots images for Nlrp3, IL-1β p17 and β-tubulin expressed in retinas of the PBS, MSC^tdTmato^ and MSC^CCR5^ groups. (D-F) Blots images for Nlrp3, IL-1β p17 and β-tubulin expressed in BV2 LPS-treated BV2 microglia cultured in the absence (PBS) or presence of MSC^tdTmato^ or MSC^CCR5^ cells. The selected parts in the red box in the figure are used for display.
