# Supplementary material for: CCR5-overexpressing mesenchymal stem cells protect against experimental autoimmune uveitis: insights from single-cell transcriptome analysis
Source: J Neuroinflammation. 2024 May 27;21:136. doi: 10.1186/s12974-024-03134-3 (PMC11131209; doi:10.1186/s12974-024-03134-3)
Supplement: Supplementary file 2 — Supplementary Material 2 [file 12974_2024_3134_MOESM2_ESM.pdf]

Supplementary Materials

Figures S1-S7 and Tables S1-S7

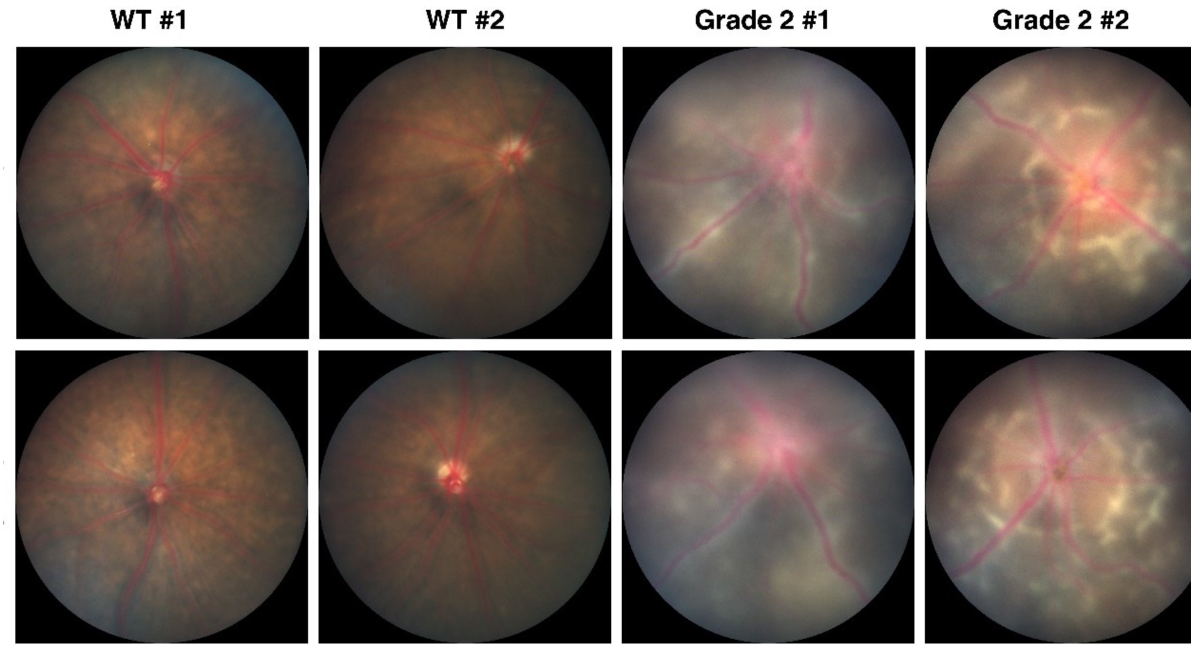

Figure S1. Fundus images of retinas used for scRNA-seq analysis. Two wild-type control mice and two grade 2 mice (14 d.p.i.) were used for scRNA-seq analysis.

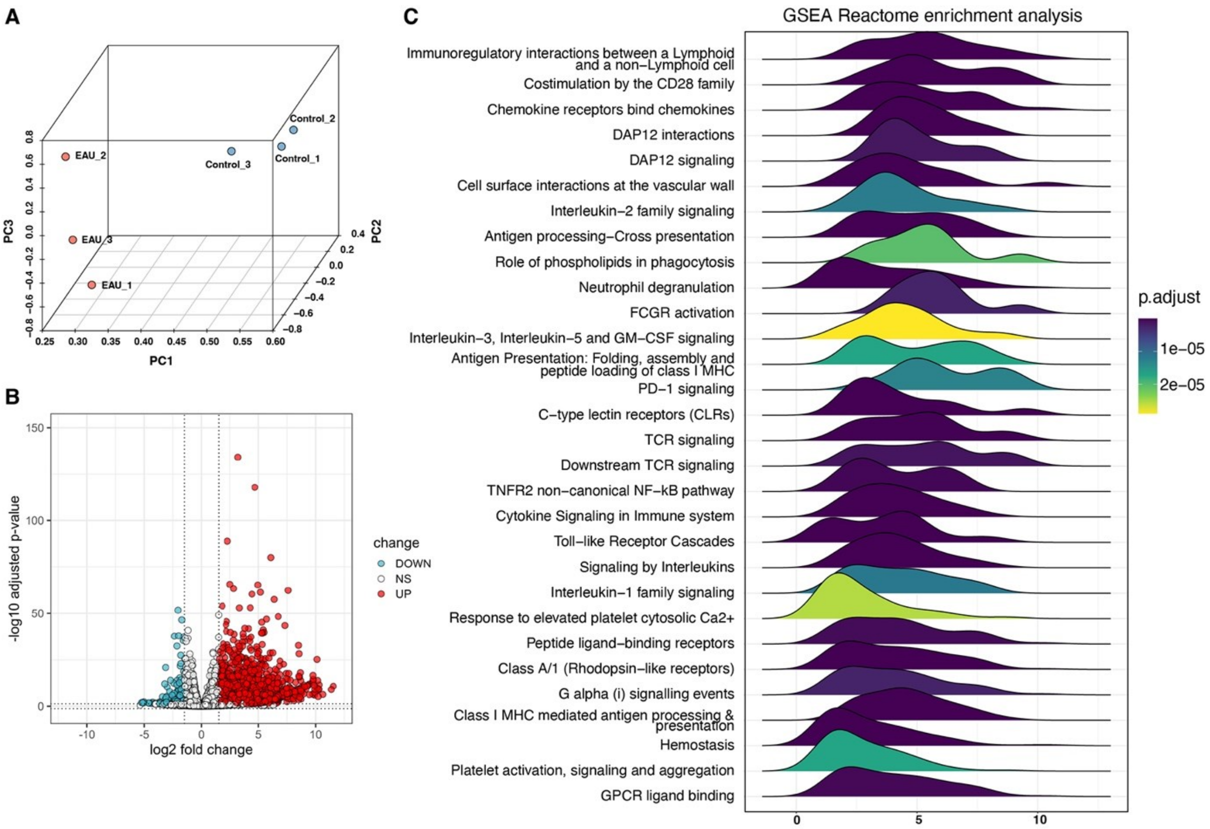

Figure S2. RNA-seq analysis of control and EAU retinas. (A) PCA (principal component analysis) plot showing that EAU retinal samples were well discriminated from the control retinal samples. (B) Volcano plot (significance vs fold change) of significantly downregulated (blue) and upregulated (red) genes (fold change $\geqslant$ 1.5 and $p < 0.05$) between EAU and control retinas. (C) GSEA Reactome pathway enrichment analysis of all known genes.

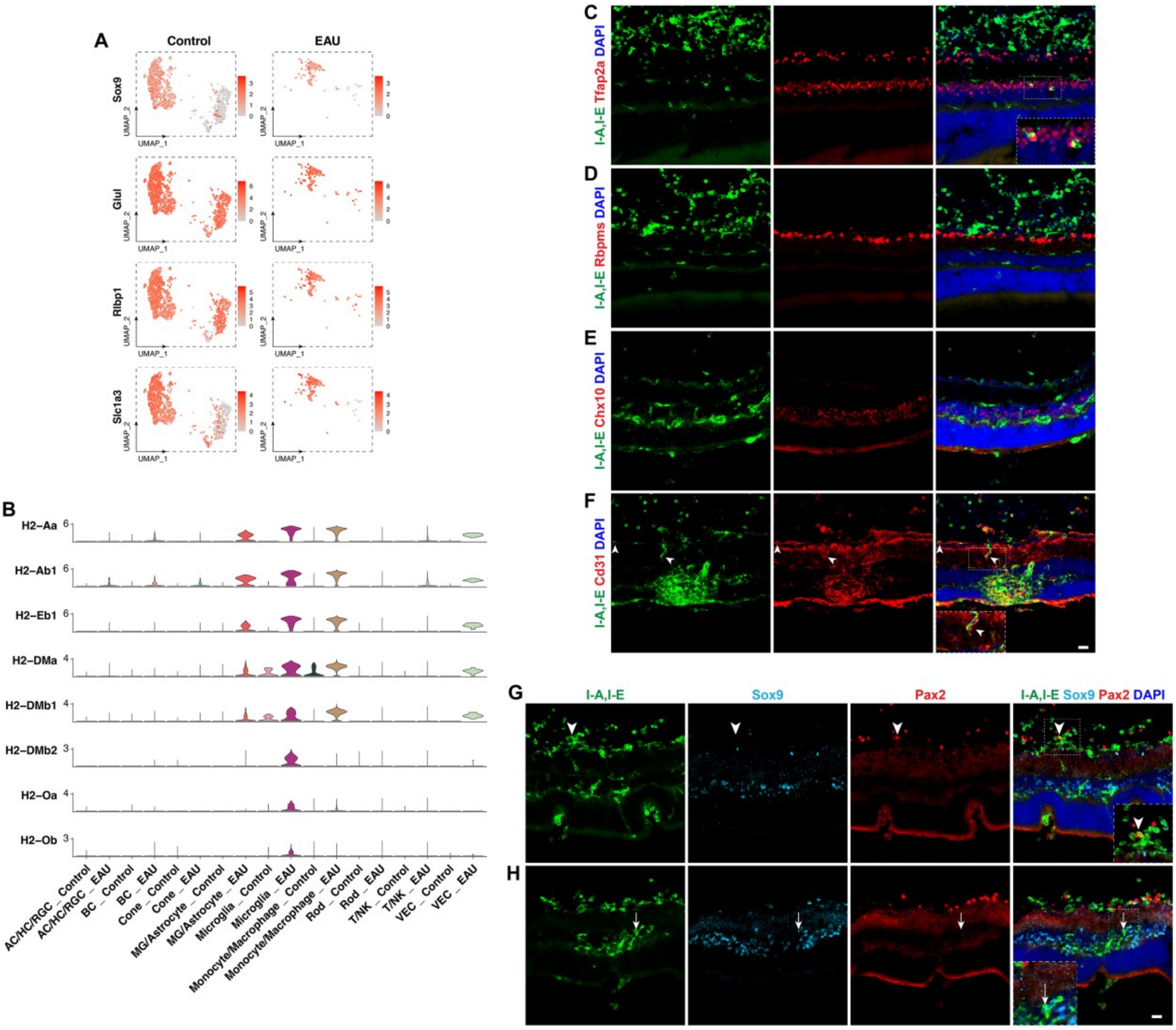

Figure S3. Expression of MHC II genes in control and EAU retinas. (A) Feature plots comparing the expression of known Müller glia marker genes in control and EAU retinas. (B) Stacked violin plots showing expression patterns of MHC-II genes in different cell types. (C) Immunostaining of EAU retinal sections for MHC II proteins I-A/I-E (green) and Tfap2a (red) with DAPI counter-labeling (blue). (D) Immunostaining of EAU retinal sections for MHC II proteins I-A/I-E (green) and Rbpms (red) with DAPI counter-labeling (blue). (E) Immunostaining of EAU retinal sections for MHC II proteins I-A/I-E (green) and Chx10 (red) with DAPI counter-labeling (blue). (F) Immunostaining of EAU retinal sections for MHC II proteins I-A/I-E (green) and Cd31 (red) with DAPI counter-labeling (blue). Arrowheads indicate representative MHC II$^+$Cd31$^+$ VECs. (G, H) Immunostaining of EAU retinal sections for MHC II proteins I-A/I-E (green), Sox9 (cyan), and Pax2 (red) with DAPI counter-labeling (blue). The arrowheads indicate a representative MHC II$^+$Pax2$^+$Sox9$^-$ astrocyte, and the arrows indicate a representative MHC II$^+$Sox9$^+$Pax2$^-$ Müller glia.

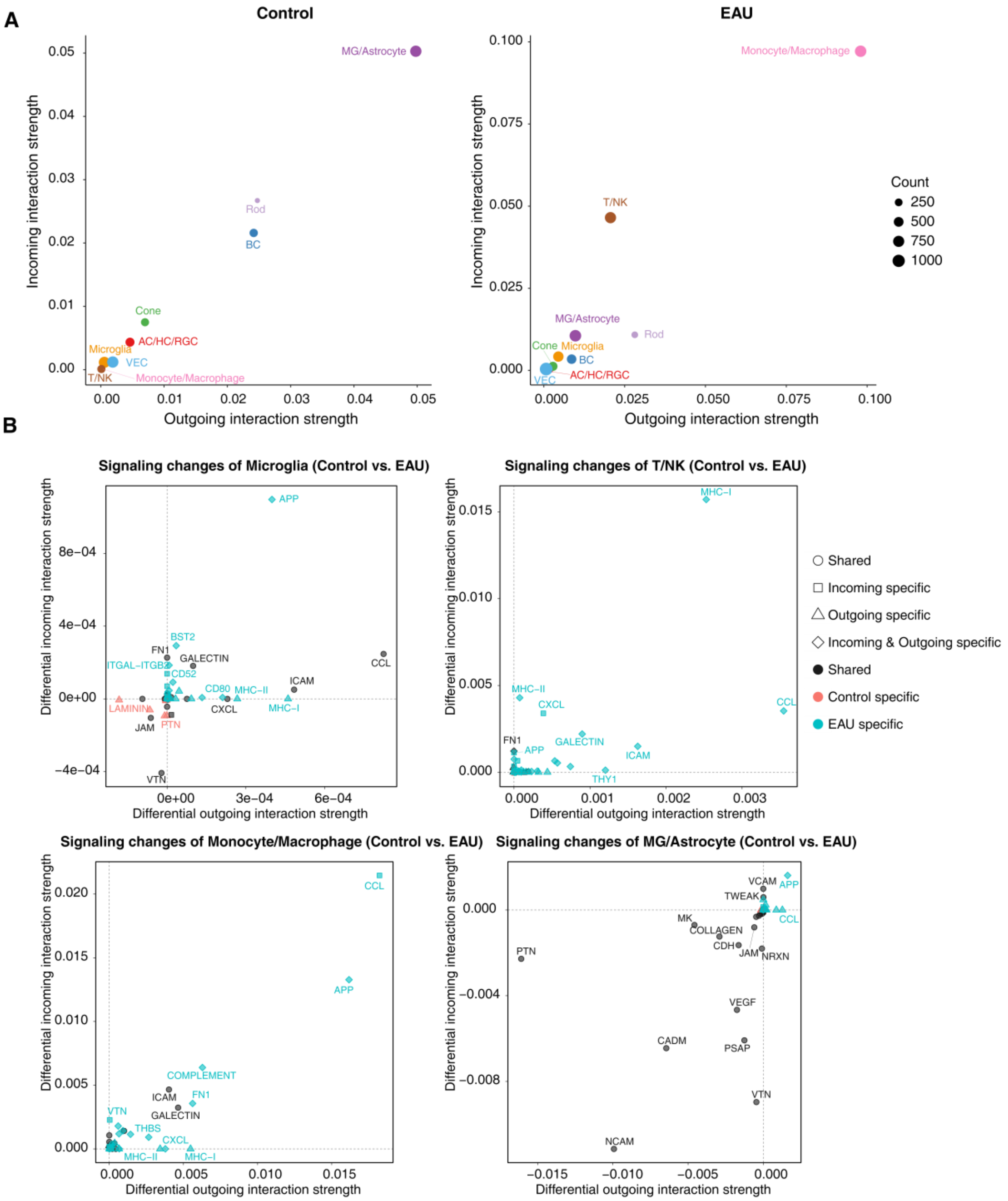

Figure S4. Changes in strength of outgoing and incoming signal interactions in EAU retinas. (A) Comparison of the strength of outgoing and incoming interactions in 2D space to identify cell populations that send or receive signals that change significantly between control and EAU retinas. (B) Visualization of differential outgoing and incoming signaling changes in Microglia, T/NK, Monocyte/Macrophage, and MG/Astrocyte cell clusters in EAU retinas compared to controls.

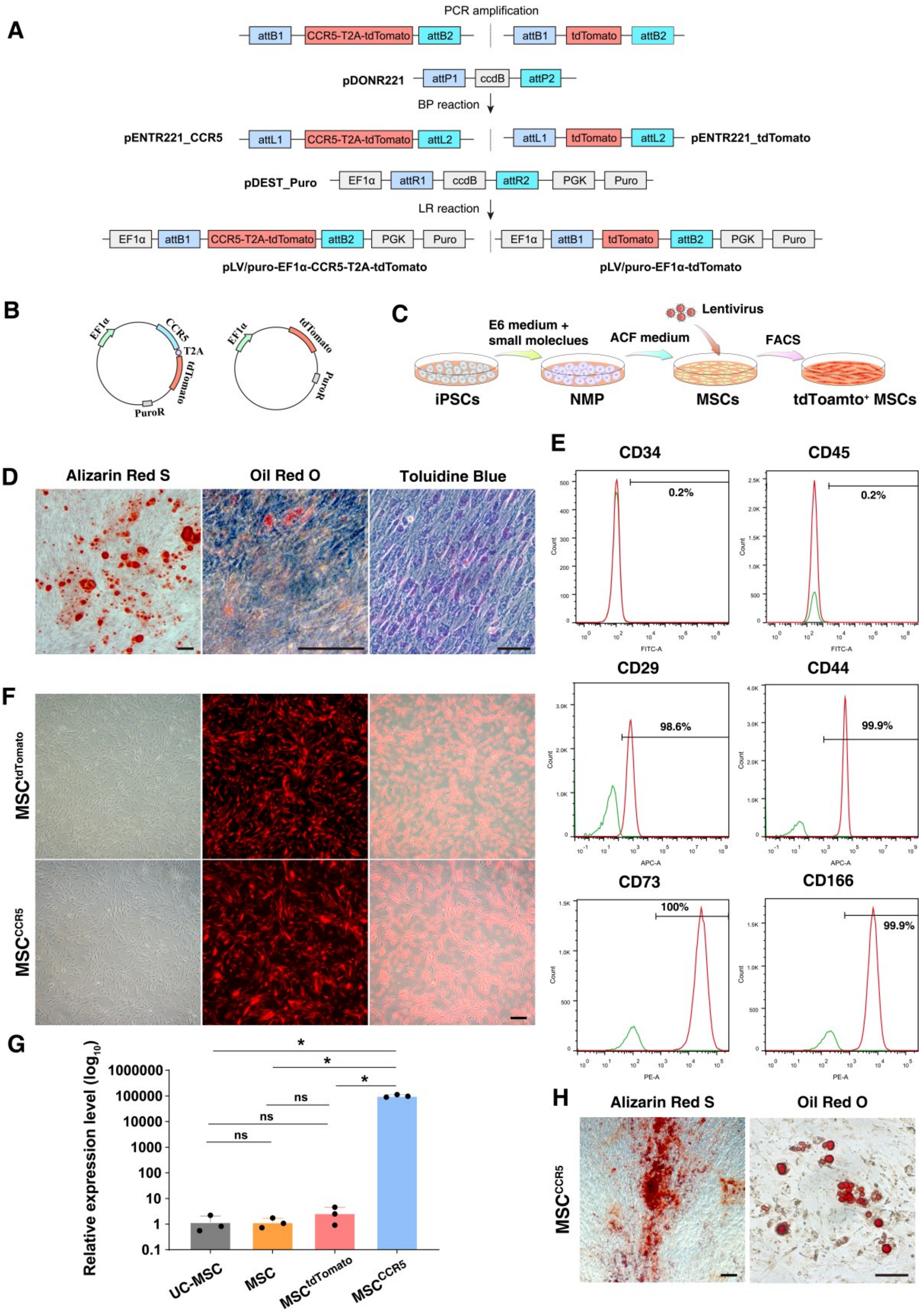

Figure S5. MSC induction and modification. (A) Schematic of the procedure for construction of the pLV/Puro-EF1α-CCR5-T2A-tdTomato and pLV/Puro-EF1α-tdTomato lentiviral expression plasmids. These plasmids were constructed using the gateway cloning

method. (B) Schematic diagrams of the pLV/Puro-EF1α-CCR5-T2A-tdTomato and pLV/Puro-EF1α-tdTomato plasmids. (C) Schematic diagram of the procedure for MSC induction from human iPSCs, lentiviral infection, and enrichment of $tdTomato^{+}$ MSCs. (D) Following differentiation, the osteogenic, adipogenic, and chondrogenic differentiation potential of hiPSC-derived MSCs were verified by staining with alizarin red S, oil red O and toluidine blue, respectively. (E) Flow cytometric analysis for detection of several typical MSC surface protein markers (CD29, CD44, CD73, and CD166) and hematopoietic markers (CD34 and CD45). (F) Normal morphologies of $MSC^{tdTomato}$ and MSC^{CCR5} cells under bright field and fluorescence microscopy (red fluorescence resulted from expressed tdTomato). (G) qRT-PCR assay of *CCR5* in UC-MSC, MSC, $MSC^{tdTomato}$ and MSC^{CCR5}. Data are presented as mean ± SD (n = 3 individual samples per group). *$p < 0.000001$, ns, no significance. (H) The osteogenic and adipogenic differentiation potential of MSC^{CCR5} cells were verified by staining with alizarin red S and oil red O, respectively. ACF: animal component-free; FACS: fluorescence-activated cell sorting; NMP: neuromesodermal progenitor; UC: umbilical cord. Scale bar: (D, F, H) 100 μm.

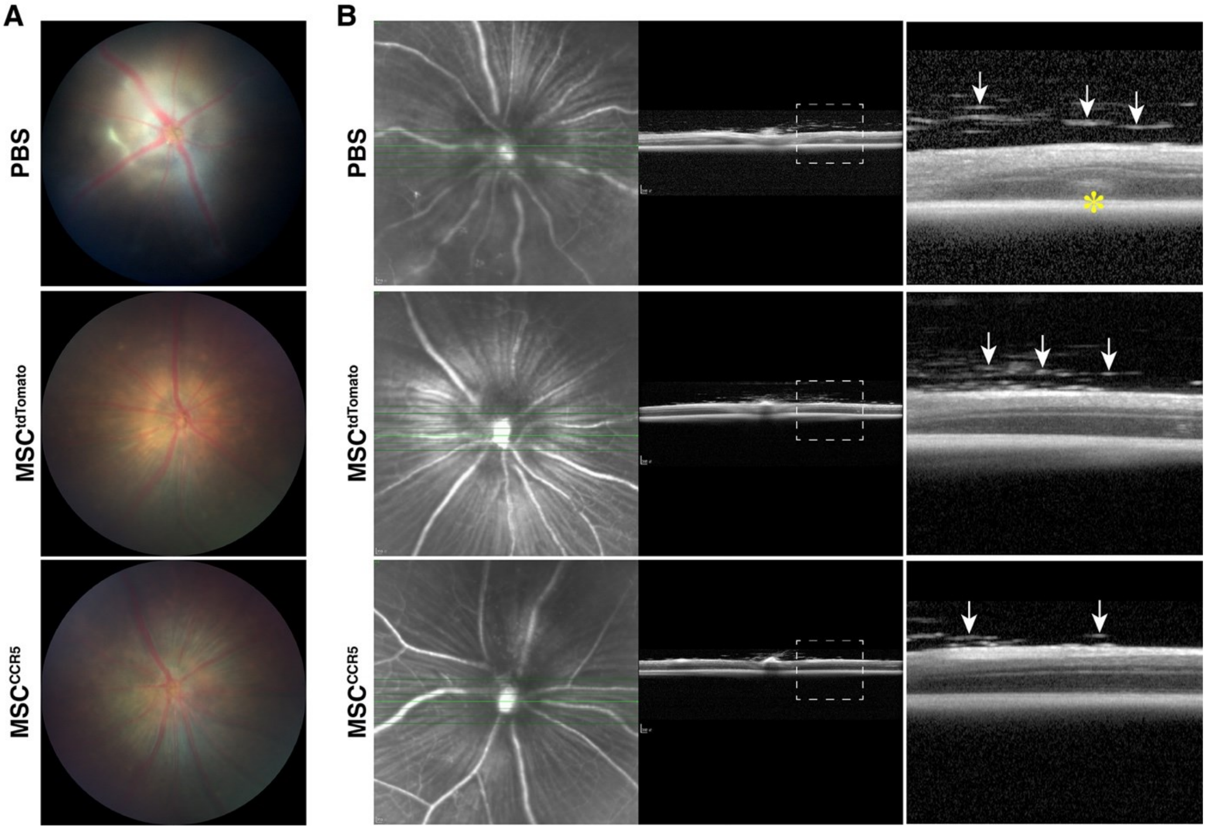

Figure S6. Transplantation of MSCs ameliorated EAU *in vivo*. (A) Representative fundus photographs of the eyes from EAU mice at 14 d.p.i., transplanted with $MSC^{tdTomato}$ or MSC^{CCR5} cells, or administered with PBS. (B) Representative fundus photographs (left) and OCT images (middle and right) of the eyes from EAU mice at 14 d.p.i., transplanted with $MSC^{tdTomato}$ or MSC^{CCR5} cells, or administered with PBS. The right panels are higher magnification views of the corresponding outlined regions in the middle panels. The arrows point to inflammatory infiltrating cells and the asterisk indicates retinal fold and detachment in EAU eyes.

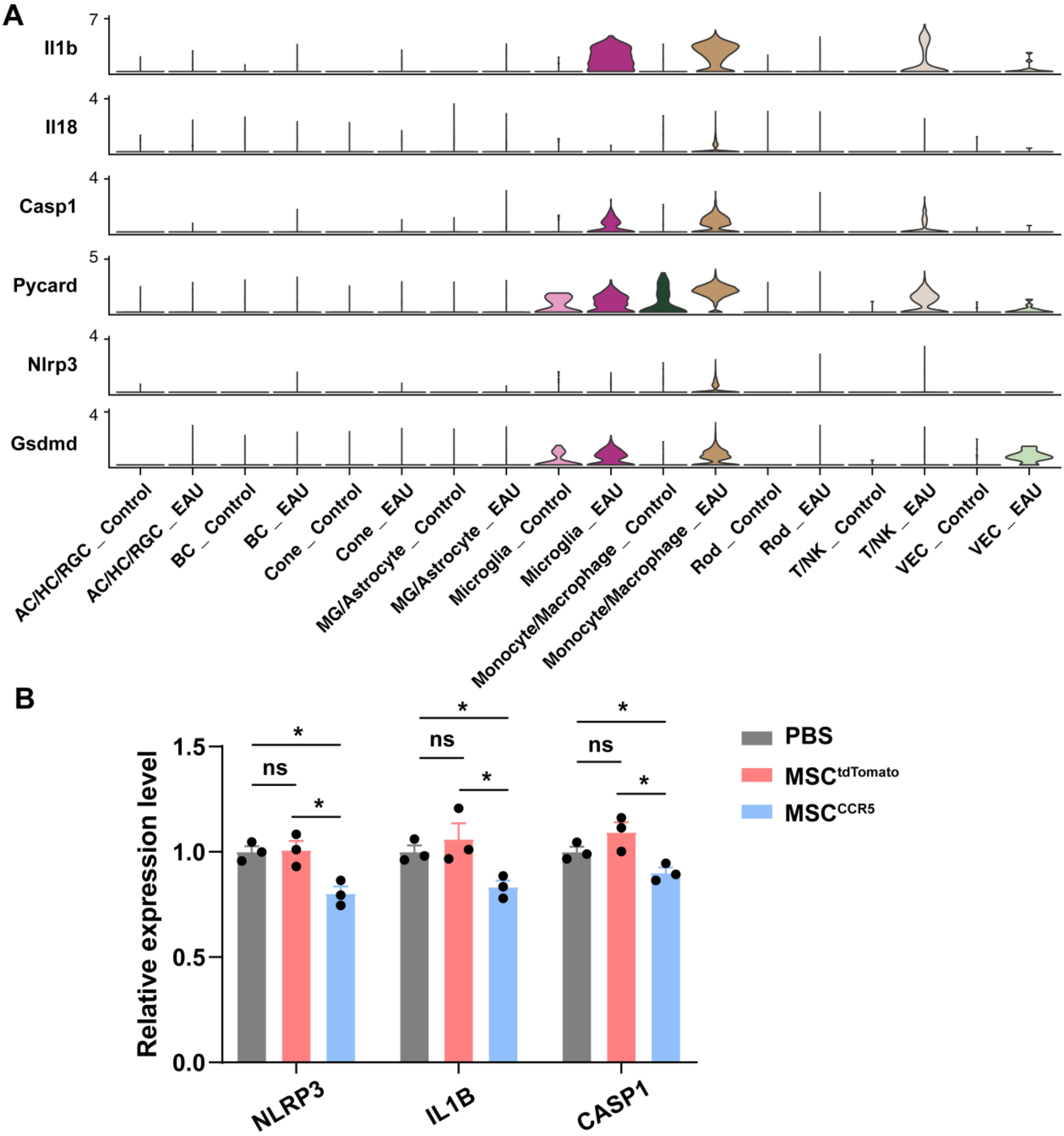

Figure S7. The NLRP3 inflammasome was activated in EAU and suppressed by MSCs. (A) Stacked violin plots showing the expression patterns of Nlrp3 inflammasome genes in control and EAU retinas based on scRNA-seq analysis. (B) qRT-PCR analysis was performed for *NLRP3*, *IL1B* (IL-1β) and *CASP1* (Caspase-1) expressed in PMA and LPS-treated THP-1 cells cultured in the absence (PBS) or presence of $MSC^{tdTomato}$ or MSC^{CCR5} cells. Data are presented as mean ± SEM (n = 3 individual samples per group). *$p < 0.05$, ns, no significance.

Table S1. Adapted clinical scoring criteria of EAU

Clinical Grade	Criteria
0	Normal retina
0.5	Few (1~2), very small and peripheral focal lesions; minimal vasculitis and vitritis
1	Mild vasculitis; <5 small, peripheral and focal lesions; 1 linear lesion
2	Retinal edema; severe vasculitis (large size, thick wall, infiltrations); diffuse chorioretinal lesions and/or infiltrations; <5 linear lesions
3	Papilledema; a pattern of linear lesions; large confluent chorioretinal lesions; subretinal neovascularization; subretinal hemorrhages
4	Large retinal detachment; retinal atrophy

Table S2. Marker genes used for identifying major retinal cell types

Retinal Cell Types	Marker genes
Amacrine cell	*Gad1, Slc6a9, Stx1b, Calb2, Chat, Th, Slc17a8, Ebf3, Tfap2a, Tfap2b*
Astrocyte	*Pax2, Gfap, Vim, S100b*
Bipolar cell	*Scgn, Grik1, Vsx1, Lhx4, Glra1, Fezf2, Zfhx4, Otx2, Vsx2, Trpm1, Grm6, Sebox, Prkca, Vstm2b*
Cone	*Opn1sw, Opn1mw, Gnat2, Arr3, Pde6h*
Horizontal cell	*Lhx1, Calb1, Gja10, Onecut1，Lim1*
Müller glia	*Abca8a, Aldh1a1, Kncj10, Sox9, S100a10, Apoe, Slc1a3, Apoe, Dkk3, Gpr37, Rlbp1, Rax, Hes1, Notch1, Glul*
Retinal ganglion cell	*Sncg, Pou4f1, Nrn1, Slc17a6, Pou4f2, Rbpms*
Rod	*Rho, Gnat1, Cnga1, Nrl, Nr2e3，Rcvrn*
Vascular endothelial cell	*Cldn5, Cdh5, Pecam1, Vwf, Erg, Tek, Kdr, Flt1*

Table S3. Marker genes used for identifying major immune cell types

Immune Cell Types	Marker genes
Microglia	*Tmem119, P2ry12, Crybb1, Rspo1, Serpine2, Astn1, Cx3cr1, Csf1r, Cd68, Aif1*
Monocytes/Macrophages	*Ccl2, Ccr2, Ly6c2, Mrc1, Itgax, Cx3cr1, Csf1r, Aif1*
T cells	*Cd3d, Cd3e, Cd3g, Il2, Cd4, Cd8a, Cd8b1, Foxp3*
Cd8 T cells	*Cd8a, Cd8b1, Cd3e*
NK cells	*Ncr1, Klra1, Klra6, Klrb1c, Klrb1a, Klrb1b, Klrb1f*
Th1 cells	*Tbx21, Cxcr6, Ifng, Tnf, Stat4, Cd4*
Th17 cells	*Il17a, Il17f, Rorc, Stat3, Cd4*
Treg cells	*Foxp3, Il2ra, Il10, Tgfb1, Cd4*
Undifferentiated T cells	*Cd3e, S1pr1, Ccr7, Il6ra, Lef1, Cd44, Cd69, Cd4*

Table S4. Gene-specific primer sequences used for qRT-PCR analysis

Gene	Forward Primer	Reverse Primer
Ccl2	CCCAAAGAAGCTGTAGTTTTTGTCA	GACCTTAGGGCAGATGCAGTT
Ccl3	CCAAGTCTTCTCAGCGCCA	CGGTTTCTCTTAGTCAGGAAAATGA
Ccl4	CTCTCCTCTTGCTCGTGGC	TGCCTCTTTTGGTCAGGAATACCA
Ccl5	CATATGGCTCGGACACCACT	TCGAGTGACAAACACGACTG
Ccl6	ATCAAGCCGGGCATCATCTTT	TTCCCAGATCTTGGGCCTTG
Ccl7	TCCCTGGGAAGCTGTTATCTTCAA	GCTATAGCCTCCTCGACCCA
Ccl8	ATGGAAGCTGTGGTTTTCCAGA	ACTTCTGGTCAAGGATCTCCA
Ccl9	CAGGCCGGGCATCATCTTTA	AGGTCCGTGGTTGTGAGTTT
Ccl12	CGGGAAGCTGTGATCTTCAGGA	TGAAGGTTCAAGGATGAAGGTTTG
Ccl17	ACTTCAAAGGGGCCATTCCT	TGGACAGTCAGAAACACGATGG
Ccl22	CAAGCCTGGCGTTGTTTTGA	TGGAGTAGCTTCTTCACCCAG
Ccl25	GCAACCTACGTGCTGTGAGA	GGCATCATCACCATCCTGGG
Ccl27a	GGAGGAGATCTACTGGGGCT	GGCAGAGGCAAGGCTTCTT
Gsdmd	GCGATCTCATTCCGGTGGACAG	TTCCCATCGACGACATCAGAGAC
Il1b (IL-1β)	TGAAATGCCACCTTTTGACAG	CCACAGCCACAATGAGTGATAC
Nlrp3	GGTCCTCTTTACCATGTGCTTC	AAGTCATGTGGCTGAAGCTGTA
Caspase1	CAGGCAAGCCAAATCTTTATCACT	GTGCCATCTTCTTTGTTCTGTTCTT
Gapdh	AGGTCATCCCAGAGCTGAACG	CACCCTGTTGCTGTAGCCGTAT
GAPDH	GGACTCATGACCACAGTCCA	TCAGCTCAGGGATGACCTTG
NLRP3	AACATGCCCAAGGAGGAAGA	GGCTGTTCACCAATCCATGA
IL1B	TGCTCAAGTGTCTGAAGCAG	TGGTGGTCGGAGATTCGTAG
CASP1	GCACACGTCTTGCTCTCATT	GCCTCCAGCTCTGTAGTCAT
CCR5	CAATGTGAAGCAAATCGCAG	GGATGAGGATGACCAGCATGT

Table S5. Antibodies used in flow cytometry

Antibodies	Supplier	Cat. No.	Host
CD29-APC	BD Biosciences	559883	Mouse
CD34-FITC	BD Biosciences	555821	Mouse
CD44-APC	BD Biosciences	559942	Mouse
CD45-FITC	BD Biosciences	555482	Mouse
CD73-PE	BD Biosciences	550257	Mouse
CD166-PE	BD Biosciences	559263	Mouse

Table S6. Primary antibodies used in western blotting and immunostaining

Primary antibodies	Supplier	Cat. No.	Host	Dilution
Nlrp3	Abcam	ab214185	Rabbit	1:500
Nlrp3	R&D system	NBP2-12446	Rabbit	1:500
Il-1β	Abcam	ab234437	Rabbit	1:500
β-Tubulin	Abcam	ab179513	Rabbit	1:1000
Sox9	Millipore	AB5535	Rabbit	1:1000
tdTomato	Sicgen	AB8181-200	Goat	1:1000
Calbindin	Swant	CB-38	Rabbit	1:7000
Chx10	Santa Cruz	sc-21690	Goat	1:1000
Tfap2a	Abcam	ab108311	Rabbit	1:1000
RBPMS	Novus Biologicals	NBP2-20112	Rabbit	1:1000
Cd45	ThermoFisher	14-0451-82	Rat	1:1000
Cd4-647	Biolegend	100533	Rat	1:500
I-A/I-E-488	Biolegend	107615	Rat	1:1000
Cd8a	ThermoFisher	14-0081-82	Rat	1:1000
Cd13/32	Abcam	ab223200	Rabbit	1:1000
Foxp3	R&D system	MAB8214-SP	Rabbit	1:1000
Cd45	R&D system	AF114-SP	Goat	1:1000
Cd11b	Abcam	ab8878	Rat	1:1000
Iba1	Abcam	ab178847	Rabbit	1:1000
Glutamine Synthetase	Millipore	MAB302	Mouse	1:1000
Tbx21	Novus Biologicals	NBP1-43299	Mouse	1:1000
Cd206	R&D System	AF2535	Goat	1:1000
Rhodopsin	Millipore	MAB5356	Mouse	1:1000
Arrestin	Millipore	AB15282	Rabbit	1:2000
F4/80	Novus Biologicals	NB600-404SS	Rat	1:400
Ccl5	Abcam	ab9679	Rabbit	1:500
Tbx21-488	Biolegend	644830	Mouse	1:500

Table S7. Secondary antibodies used in Western blotting and immunostaining

Secondary antibodies	Supplier	Cat. No.	Dilution
Goat anti-Rabbit HRP-linked IgG	Abcam	ab6721	1:5000
Horse anti-Mouse HRP-linked IgG	Cell Signaling Technology	7076	1:2000
Donkey anti-Mouse Alexa 594 IgG	Invitrogen	A-21203	1:1000
Donkey anti-Mouse Alexa 488 IgG	Invitrogen	A-21202	1:1000
Donkey anti-Goat Alexa 594 IgG	Invitrogen	A-11058	1:1000
Donkey anti-Rabbit Alexa 594 IgG	Invitrogen	A-21207	1:1000
Donkey anti-Rabbit Alexa 488 IgG	Invitrogen	A-21206	1:1000
Donkey anti-Rat Alexa 647 IgG	Invitrogen	A-78947	1:1000
Donkey anti-Rat Alexa 594 IgG	Invitrogen	A-21209	1:1000
Donkey anti-Rat Alexa 488 IgG	Invitrogen	A-21208	1:1000
